# Polymorphism in Mitochondrial Group I Introns among *Cryptococcus neoformans* and *Cryptococcus gattii* Genotypes and Its Association with Drug Susceptibility

**DOI:** 10.3389/fmicb.2018.00086

**Published:** 2018-02-06

**Authors:** Felipe E. E. S. Gomes, Thales D. Arantes, José A. L. Fernandes, Leonardo C. Ferreira, Héctor Romero, Sandra M. G. Bosco, Maria T. B. Oliveira, Gilda M. B. Del Negro, Raquel C. Theodoro

**Affiliations:** ^1^Department of Biochemistry, Universidade Federal do Rio Grande do Norte, Natal, Brazil; ^2^Institute of Tropical Medicine of Rio Grande do Norte, Universidade Federal do Rio Grande do Norte, Natal, Brazil; ^3^Laboratorio de Organizacion y Evolución del Genoma/Unidad de Genómica Evolutiva, Departamento de Ecología y Evolución, Facultad de Ciencias/CURE, Universidad de la República, Maldonado, Uruguay; ^4^Department of Microbiology and Immunology, Institute of Biosciences, Universidade Estadual Paulista Julio de Mesquita Filho, São Paulo, Brazil; ^5^Department of Microbiology and Parasitology, Universidade Federal do Rio Grande do Norte, Natal, Brazil; ^6^Institute of Tropical Medicine of São Paulo, Universidade de São Paulo, São Paulo, Brazil

**Keywords:** group I introns, mtDNA, *LSU*, *Cryptococcus* genotypes, antifungal susceptibility, 5-fluorocytosine, homing endonuclease

## Abstract

Cryptococcosis, one of the most important systemic mycosis in the world, is caused by different genotypes of *Cryptococcus neoformans* and *Cryptococcus gattii*, which differ in their ecology, epidemiology, and antifungal susceptibility. Therefore, the search for new molecular markers for genotyping, pathogenicity and drug susceptibility is necessary. Group I introns fulfill the requisites for such task because (i) they are polymorphic sequences; (ii) their self-splicing is inhibited by some drugs; and (iii) their correct splicing under parasitic conditions is indispensable for pathogen survival. Here, we investigated the presence of group I introns in the mitochondrial *LSU rRNA* gene in 77 *Cryptococcus* isolates and its possible relation to drug susceptibility. Sequencing revealed two new introns in the *LSU rRNA* gene. All the introns showed high sequence similarity to other mitochondrial introns from distinct fungi, supporting the hypothesis of an ancient non-allelic invasion. Intron presence was statistically associated with those genotypes reported to be less pathogenic (*p* < 0.001). Further virulence assays are needed to confirm this finding. In addition, *in vitro* antifungal tests indicated that the presence of *LSU rRNA* introns may influence the minimum inhibitory concentration (MIC) of amphotericin B and 5-fluorocytosine. These findings point to group I introns in the mitochondrial genome of *Cryptococcus* as potential molecular markers for antifungal resistance, as well as therapeutic targets.

## Introduction

Cryptococcosis is a systemic mycosis that affects humans and animals. Humans are often infected by inhalation of infective propagules, which first colonize the lungs and subsequently invade the central nervous system (Chen et al., 2014). Currently, two species are recognized as etiological agents of this disease: *Cryptococcus neoformans* and *Cryptococcus gattii* (Kwon-chung et al., 2014). However, a recent study using multi-loci analysis suggested the division of *C. neoformans*/*C. gattii* into seven different species (Hagen et al., 2015). Nevertheless, for clinical practicality, the names *C. neoformans* and *C. gattii* species complexes are used.

Various molecular techniques have already been applied to the epidemiological study of cryptococcosis (Brandt et al., 1995; Yamamoto et al., 1995; Boekhout et al., 2001; Meyer et al., 2003, 2009; Leal et al., 2008), resulting in the recognition of eight genotypes: VNI (serotype A), VNII (serotype A), VNIII (hybrid AD), VNIV (serotype D) for *C. neoformans*, and VGI, VGII, VGIII, VGIV (serotypes B and C) for *C. gattii*.

These genotypes differ in many aspects. Ecologically, *C. neoformans* is known to be associated with feces of pigeons (*Columba livia*), and other birds, whereas *C. gattii* is frequently isolated from vegetal material, such as *Eucalyptus* trees (Sorrell, 2001; Gullo et al., 2013). Epidemiologically, for many years infections caused by *C. neoformans* were considered the major cause of morbidity and mortality in immunosuppressed patients (mainly those with AIDS), while *C. gattii* usually infects immunocompetent patients (Sorrell, 2001; Iqbal et al., 2010; D'Souza et al., 2011). Although all the *C. gattii* genotypes are able to cause disease as primary pathogens, VGI and VGII were shown to affect immunocompetent individuals more frequently than VGIII and VGIV (Farrer et al., 2015). Some genotype-associated clinical differences have also been observed; for example, VGI infection usually affects the central nervous system, presenting cryptococcomas and simultaneous lung lesions, whereas VGII generally causes pulmonary disease. These differences require refined therapeutic recommendations specific for the causative *C. gattii* genotype (Chen et al., 2014). The genotypes also show geographic variation; VGII and VNI are frequently found in the Americas, VGIV predominates in the southern countries of Africa, and VGI and VNIV occur in Europe (Matos et al., 2012; Chen et al., 2014; Kwon-chung et al., 2014). Finally, antifungal responses may also vary. In some studies, *C. gattii* disease has been reported to present longer clinical courses (delayed clinical and mycological cure) than *C. neoformans* (Sorrell, 2001). Additionally, the molecular type VGII is less susceptible to antifungal drugs (especially azoles), followed by VGI, VNI, and VNIV (Chong et al., 2010; Hagen et al., 2010; Iqbal et al., 2010; Trilles et al., 2012). Thus, the *Cryptococcus* species, genotype and geographic origin are important data that must be taken into consideration for choosing the correct treatment (Chong et al., 2010; Trilles et al., 2012).

Although the methods for molecular differentiation are very specific, their application is time-consuming, normally requires multiple steps and equipment, and in some cases, depends on subjective interpretation, such as the methodologies involving sequencing (Meyer et al., 2009), multiplex PCR (Leal et al., 2008), AFLP (Boekhout et al., 2001) and RFLP (Meyer et al., 2003). Therefore, new polymorphic DNA markers, with the potential for a practical and reproducible indication of drug resistance/susceptibility among *Cryptococcus* genotypes, are important for clinical and epidemiological studies. Thus, we evaluated the group I introns in the mitochondrial large subunit *rRNA* gene (*LSU*) as a possible candidate for this purpose.

Group I introns are structural sequences capable of catalyzing their own splicing from precursor RNA (Lambowitz and Caprara, 1999; Haugen et al., 2005). Some of them are mobile elements due to the presence of homing endonucleases, which trigger a DSB (Double Strand Break), usually in an allelic site, that is followed by homologous recombination repair (Belfort and Roberts, 1997; Stoddard, 2011). These introns are also important drug targets because once the splicing is inhibited, the precursor RNA remains non-functional. In *Candida albicans*, for example, the nuclear group I intron in the Ca.*LSU rRNA* gene is related to increased susceptibility to pentamidine (Miletti and Leibowitz, 2000; Zhang et al., 2002) and bleomycin (Jayaguru and Raghunathan, 2007), since these drugs selectively inhibit group I intron self-splicing.

The search for new therapeutic targets is urgent due to the limited availability of antifungal agents in the face of the increasing occurrence of opportunistic fungal infections and drug resistant strains (Dismukes, 2000; Singh, 2001; Cowen et al., 2014). In addition, the antifungals commonly used in cryptococcosis treatment, such as amphotericin B, can cause serious toxic side effects in patients, usually requiring hospitalization (Laniado-Laborín and Cabrales-Vargas, 2009). In this sense, group I introns may be considered a safe therapeutic target because they are absent in the human genome (Disney et al., 2001).

The present study was designed to investigate the occurrence and variability of mitochondrial *LSU rRNA* introns in *C. neoformans* and *C. gattii*, and their possible relationship to genotype and antifungal susceptibility. Two group I introns (Cne.mL2449 and Cne.mL2504) in the mitochondrial *LSU rRNA* of *Cryptococcus* have already been described in a *C. neoformans* var. *neoformans* (VNIII genotype) isolate (Cannone et al., 2002; Litter et al., 2005). In this work we also propose the nomenclature and structure for two newly found *LSU rRNA* group I introns in *C. neoformans* and *C. gattii*. Our main findings indicate that the *LSU rRNA* intronless genotypes are those reported to be the most virulent ones (especially VGII, VGI, and VNI) and also that *in vitro* antifungal tests indicate a relationship between intron absence and high MIC (minimum inhibitory concentration) values for 5-fluorocytosine.

## Materials and methods

### Isolates used in this work

Seventy-seven *C. neoformans* and *C. gattii* isolates from different sources and one isolate of their sister species, *C. laurentii*, were included in the study (Supporting Information, Table S1). Most isolates came from the mycological collection of IBB-UNESP, Botucatu, SP, Brazil, and from recent isolations of cerebrospinal fluid from patients admitted at Giselda Trigueiro Hospital, and other hospitals in Natal/RN (Brazil) (samples provided by the routine microbiological diagnostic analyses carried out at LACEN-RN - Central Laboratory of RN State). The work was conducted under the approval of the Ethics Committee of the Federal University of Rio Grande do Norte: protocols 39640614.8.0000.5537 and 45188515.1.0000.5537. Some isolates were also kindly provided by Dr. Fernanda Fonseca and Dr. Marilene Henning Vainstein at the Federal University of Piauí (Brazil) - UFPI and Federal University of Rio Grande do Sul (Brazil) – UFRGS, respectively. Reference strains were provided by FIOCRUZ-RJ (National Institute of Infectious Diseases Evandro Chagas). All isolates were maintained on Sabouraud Dextrose Agar medium with chloramphenicol (50 mg/l), at 37°C for 72 h before DNA extraction.

### DNA extraction and genotyping

DNA extraction was performed according to Trilles et al. (2008). Molecular types were determined by RFLP analysis of the PCR product of *URA5* gene. The *URA5* amplification was performed in a final volume of 50 μl. Each reaction contained 27 μL of nuclease free water (Sigma), 10 μL PCR buffer CG 5X (200 mM tris-HCL pH 8.4; 1.5 mM MgCl_2_ 50 mM; 500 mM KCl), 5 μL 30% DMSO (Thermo Scientific), 1 μL deoxynucleoside triphosphates (10 mM each, New England BioLabs), 30 ng of genomic DNA, 1 U taq *Phusion DNA Polymerase* (Finnzymes), and 1.25 μL of each primer (20 μM) *URA5* (5′-ATGTCCTCCCAAGCCCTCGACTCCG-3′) and *SJO1* (5′-TTAAGACCTCTGAACACCGTACTC-3′) (Meyer et al., 2003). PCR was carried out with an initial denaturation step at 98°C for 1 min, followed by 35 cycles of denaturation at 98°C for 30 s, annealing at 61°C for 30 s, and extension at 72°C for 1 min and then a final extension at 72°C for 10 min. PCR products were concentrated to 12.5 μL and double digested with the endonucleases *Sau96I* (5 U/μl, New England BioLabs) and *HhaI* (20 U/μl, New England BioLabs) for 3 h at 37°C, and the fragments were separated by 3% agarose gel electrophoresis stained with ethidium bromide at 100 V during 90 min. The isolates were classified following the *URA5*-RFLP pattern proposed by Meyer et al. (2003) for the eight genotypes.

### PCR and sequencing of mitochondrial LSU rRNA group I introns of *C. neoformans* and *C. gattii*

PCR of *LSU rRNA* group I Introns was performed with the primers Cry*LSU*F (5′ GATTTGACTATTCTTATGTGC 3′) and Cry*LSU*R (5′ GGTATATGCATGCTTGACTGC 3′) herein designed for annealing at 5′ and 3′ positions that flank the two introns previously described in the mitochondrial *LSU rRNA* gene (Litter et al., 2005). The concentration of each reagent used in this PCR was the same used for *URA5* amplification and the thermal cycling conditions were: initial denaturation at 98°C for 2 min, followed by 35 cycles of denaturation at 98°C for 45 s, annealing at 60°C for 1 min, extension at 72°C for 1 min and 30 s, and a final extension at 72°C for 10 min. PCR products were visualized by agarose gel electrophoresis (1%) stained with ethidium bromide at 90 V during 60 min.

The different-sized amplified DNAs of 20 isolates were purified with the *IlustraTM GFXTM PCR DNA and Gel Band Purification* kit (GE Healthcare), following the manufacturer's instructions and sequenced at MACROGEN/South Korea. All the sequences were aligned together with reference sequences (NCBI accession numbers AY560611, DQ479323, and AY560612) using PRANK online (EMBL-EBI) (http://www.ebi.ac.uk/goldman-srv/webPRANK/) (Löytynoja and Goldman, 2010). A search for homing endonuclease genes (HEG) was carried out for each sequenced intron using the Conserved Domain Database of NCBI (http://www.ncbi.nlm.nih.gov/Structure/cdd/wrpsb.cgi) (Marchler-Bauer et al., 2015). All regions showing high similarity with HEG had their sequences removed from the introns and were not included in the phylogenetic analysis.

### Phylogenetic analysis

For comparative purposes, a blastn (Basic Local Alignment Search Tool) (Altschul et al., 1990) was carried out in the GenBank database of NCBI (https://blast.ncbi.nlm.nih.gov/Blast.cgi), using the four representative introns sequenced in this study. Sequences with query cover lower than 25% were not included in the study. Sequences were aligned with PRANK on-line (EMBL-EBI) (http://www.ebi.ac.uk/goldman-srv/webPRANK/) (Löytynoja and Goldman, 2010). After alignment, a block within the intron sequence with enough genetic information for phylogenetic analysis was manually selected, excluding the peripheral nucleotides, whose sequences correspond to those helices and domains less conserved among group I introns. Thus, the greater part of the intronic sequences used in our phylogenetic analysis correspond to helices P7 and P7′ as well as their internal sequences, since that region represents the catalytic core, which is more conserved among group I introns.

Phylogenetic analyses were carried out with MrBayes v.3.2.6 (Ronquist et al., 2012). Eight independent runs, with 16 chains (15 hot and 1 cold) and 10,000,000 generations each, were carried out under an independent substitution model. Convergence was assessed by analyzing the standard deviation between runs, which remained below 0.01. In addition, after summarizing the statistical values of the run, it was verified that the values of PSRF (Potential Scale Reduction Factor) always remained very close to 1.0 for the TL, pi (A), pi (C), pi (G), and pi (T) parameters. The generated trees were summarized and a consensus tree was generated. After visualization in FigTree v.1.4.3 (Rambaut, 2009), the phylogenetic tree was exported to Inkscape (v 0.91) for additional editing, always preserving the scale.

### Sequence analysis of the newly discovered introns in the mitochondrial LSU rRNA gene of *C. neoformans* and *C. gattii*

To examine in detail the two new group I introns found in our sequencing reactions, a two-step procedure was followed. First, we used infernal v1.1.2 (Nawrocki and Eddy, 2013), to align the introns with a RFAM (Nawrocki et al., 2015) model for group I introns (RF00028) and evaluate its significance. Then, a prediction of the secondary structure was carried out by using the *mfold* RNA online software (http://unafold.rna.albany.edu/?q=mfold/RNA-Folding-Form) (Zuker, 2003). The constraints and conserved domains for group I introns as well as other conventions and proposals already reproduced by other authors (Michel and Westhof, 1990; Li and Zhang, 2005; Hausner et al., 2014) were considered. For a better localization of the P1 and P10 helices, 10 nucleotides of the flanking exon at both the 5′ and 3′ ends of the intron were included in the analysis. Introns were named according to the nomenclature proposed by the guide for group I introns in rDNA (Johansen and Haugen, 2001). The predicted structure was edited using Inkscape software (v 0.91).

### Antifungal susceptibility of cryptococcus isolates with and without group I introns in mitochondrial LSU rRNA

The *in vitro* broth microdilution method was carried out using the Clinical and Laboratory Standards Institute (CLSI), guides M27-A3 and M27-S3 (CLSI-Clinical Laboratory Standards Institute, 2008a,b). Isolates with and without introns (twenty isolates for each group) were tested with the antifungal drugs itraconazole (Sigma-Aldrich), amphotericin B (Sigma-Aldrich) and 5-fluorocytosine (Sigma-Aldrich). The stock solutions were prepared in DMSO for itraconazole and in sterile deionized Milli-Q water for 5-fluorocytosine at 1,600 μg mL^−1^ and 640 μg mL^−1^, respectively. The amphotericin B used was already in solution at 250 μg mL^−1^. Serial dilutions were prepared in RPMI 1640 with glutamine, without bicarbonate (Gibco, Life Technologies), pH 7.6 (buffered with 1M HCl) and sterilized by filtration. Drug dilutions were dispensed in 96-well microdilution plates, with the final concentrations of the drugs ranging from 0.125 to 64 μg mL^−1^ for 5-fluorocytosine and 0.03125 to 16 μg mL^−1^ for amphotericin B and itraconazole. Final inoculum concentrations ranged from 0.5 × 10^3^ to 2.5 × 10^3^ CFU/mL in each well containing different concentrations of the antifungal drugs. The quality-control strains, *Candida parapsilosis* (ATCC 22019) and *Candida krusei* (ATCC 6258), were used in each test. Drug-free and yeast-free controls were included. Plates were incubated at 35°C up to 72 h.

MICs (minimum inhibitory concentrations) for all strains were read visually 48 and 72 h after yeast inoculum. MIC for amphotericin B was defined as the lowest concentration that caused 100% inhibition of growth compared to the drug-free control well, and MICs for fluconazole and itraconazole as the lowest drug concentration that caused a prominent decrease in growth (50%) compared with the controls.

### Statistics

Intron (i.e., present or absent) and species (i.e., *C. neoformans* or *C. gattii*) data were treated as dichotomous variables. Fisher exact tests were used to test for differences in intron distribution between genotype groups (i.e., virulent vs. non-virulent, according to literature). MIC was treated as ordered variable, since it was divided into four categories from lowest to highest MIC. Thus, to assess the effect of introns on fungi response to antifungals drugs, we applied ordered logistic models using MIC as response variable and intron (absent as reference) and species information (*C. gattii* as reference) as predictors. In addition, we tested the hypothesis that the species modifies the effect of the introns on MIC by including the interaction term (introns^*^species). We used the minimum Akaike information criterion (AIC) approach to evaluate the model that better fits the data. The predicted probabilities of being in one of the MIC categories were calculated via post estimation command. The statistical analyses were performed in Stata 11.1 (StataCorp, 2017).

## Results

### Size polymorphism of PCR products from mitochondrial LSU group I introns among *C. neoformans* and *C. gattii* genotypes

According to PCR-RFLPs of the *URA5* gene, most of the 77 isolates were identified as *Cryptococcus neoformans* (*n* = 51), followed by *C. gattii* (*n* = 26). Overall, the most common molecular types were VNI (39 isolates) and VGII (20 isolates), followed by VNIV (5 isolates), VNII (4 isolates), VNIII (3 isolates) and two isolates each for VGI, VGIII, and VGIV (Supporting Information, Table S1).

**Figure 1:**
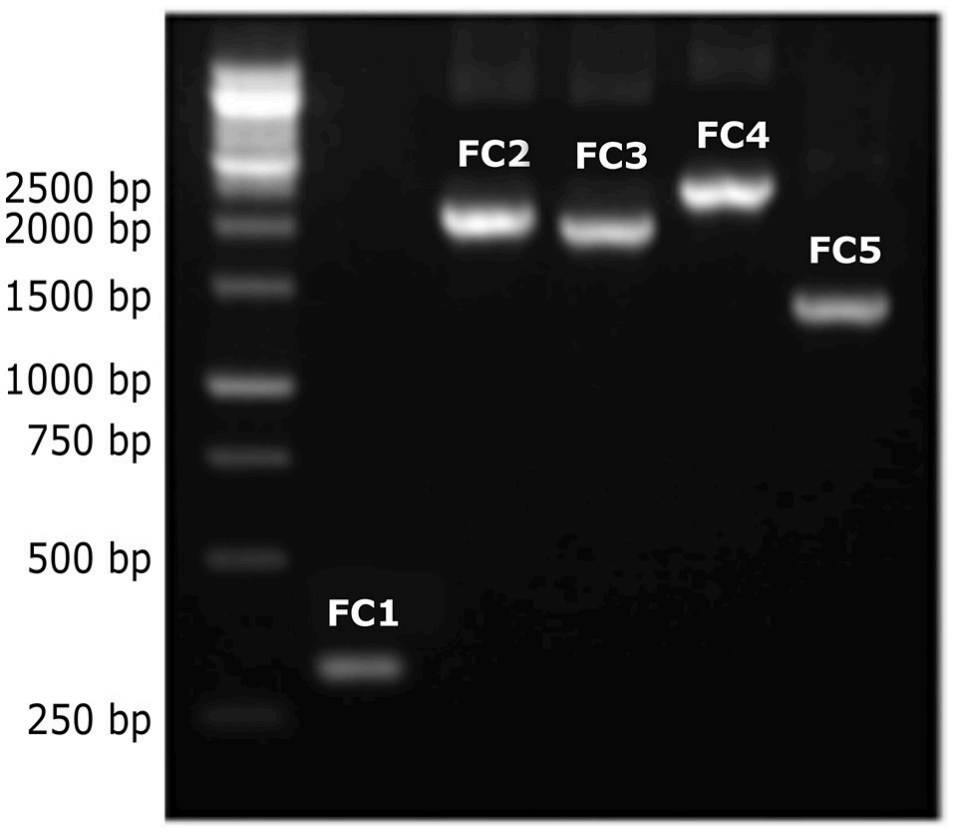
Different-sized PCR products of the *LSU rRNA* introns from *C. neoformans* and *C. gattii* genotypes. PCR products of different sizes were observed after amplification of DNA from the different molecular types using the Cry*LSU*F and Cry*LSU*R primers, followed by agarose gel electrophoresis (1%) stained with ethidium bromide. First lane: 1 kb molecular ladder (Kasvi), followed by FC1–FC5, reference isolates belonging to genotypes VGII, VNIV, VGIII, VNIII, and VNII, respectively.

**Figure 2:**
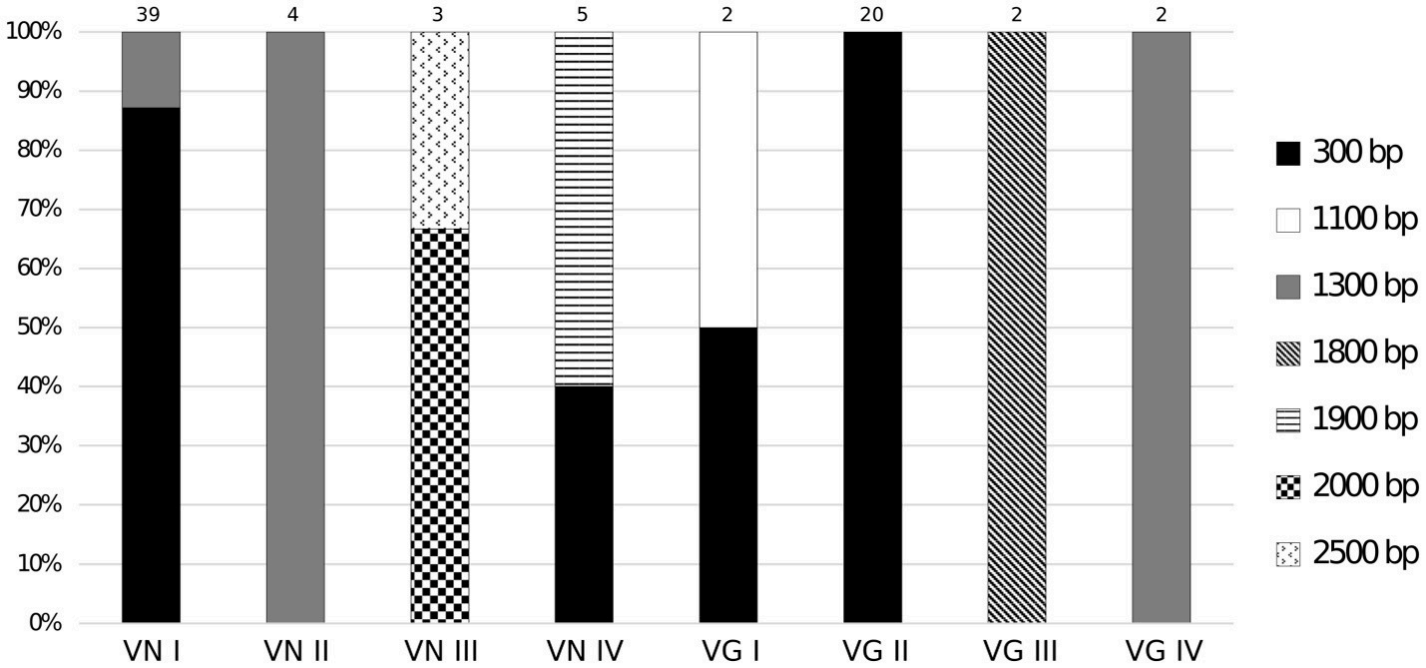
Intron PCR-size variation of the mitochondrial *LSU rRNA* gene among different genotypes of *C. neoformans* and *C. gattii*. The size polymorphisms for group I intron PCR products were associated with the molecular type. PCR-product sizes for each genotype are indicated. The 300 bp fragment indicates absence of any intron elements.

In the CRW database (Cannone et al., 2002), the mitochondrial *LSU rRNA* gene of *C. neoformans* var. *neoformans* contains two introns named Cne.mL2449 (with 1,168 bp) and Cne.mL2504 (with 417 bp) (http://www.rna.icmb.utexas.edu/). The primers Cry*LSU*F and Cry*LSUR*, herein designed, anneal to the 5′ and 3′ exons flanking these introns. The amplicon is expected to be 300 bp when the introns are absent, and around 1.9 Kb when both introns are present. It was observed that the PCR products ranged from approximately 300 bp to 2.5 Kb among the eight molecular types (Figure 1). Among the 39 VNI isolates, the 300 bp PCR product was detected in 34 of them (except for some isolates that showed a PCR product of 1.3 kb). All 20 VGII isolates, one of the two VGI isolates and two of the five VNIV isolates also yielded the 300 bp product, indicating intron absence. Among the intron-containing isolates, other sizes than 1.9 Kb were observed, such as 1.1 Kb (for some VGIs), 1.3 Kb (for some VNIs, VNIIs, and VGIVs), 1.8 Kb (for VGIII) and 1.9 Kb (for some VNIVs). The VNIII molecular type had a 2 Kb product for 2 isolates and a 2.5 Kb product for one isolate (Supporting Information, Table S1, Figure 2). *C. laurentii* showed no PCR amplification, probably due to the absence of complementary sequences to the primers herein designed.

### Description of two new introns in mitochondrial LSU rRNA in *C. neoformans* and *C. gattii*

**Figure 3:**
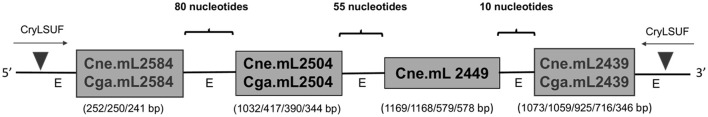
Distribution of group I introns in mitochondrial *LSU rRNA* of *C. neoformans* and *C. gattii* isolates. The scheme shows the order of the introns within the *LSU rRNA* gene observed after alignment of the sequenced samples. Introns are separated by conserved regions of the mitochondrial genome. Triangles indicate the annealing regions of the Cry*LSU*F and Cry*LSU*R primers flanking all the introns. The different sizes for each intron as well as the exon (“E”) regions are shown in the figure.

Aligned sequences of the PCR products for mitochondrial *LSU rRNA* introns of different sizes (Supporting Information, Data Sheet 1) showed that some genotypes had zero, one, two, three or four introns in this gene, and that the exons between them are very short (10, 55, and 80 bp) (Figure 3) (GenBank accession numbers KY748211-KY748230). The 300-bp sequenced fragment had 99% similarity with *LSU rRNA* gene of *C. neoformans* var. *grubii* (GenBank accession number AY560612).

The new introns, not described yet in literature for the *C. neoformans*/*C. gattii* species complex, were herein named Cne.mL2439 and Cne.mL2584, in *C. neoformans*, and Cga.mL2439 and Cga.mL2584, in *C. gattii*. Cne.mL2584/Cga.mL2584 were found in VNIII, VNIV, VGI, and VGIII genotypes whereas Cne.mL2439/Cga.mL2439 were found in all *C. neoformans*, except in VNI and in all *C. gattii*, excluding VGII. We also observed that some *C. gattii* genotypes contain a group I intron at position 2504, identifying it as Cga.mL2504. This intron was previously described only in *C. neoformans* strains (Cne.mL2504) (Litter et al., 2005).

**Figure 4:**
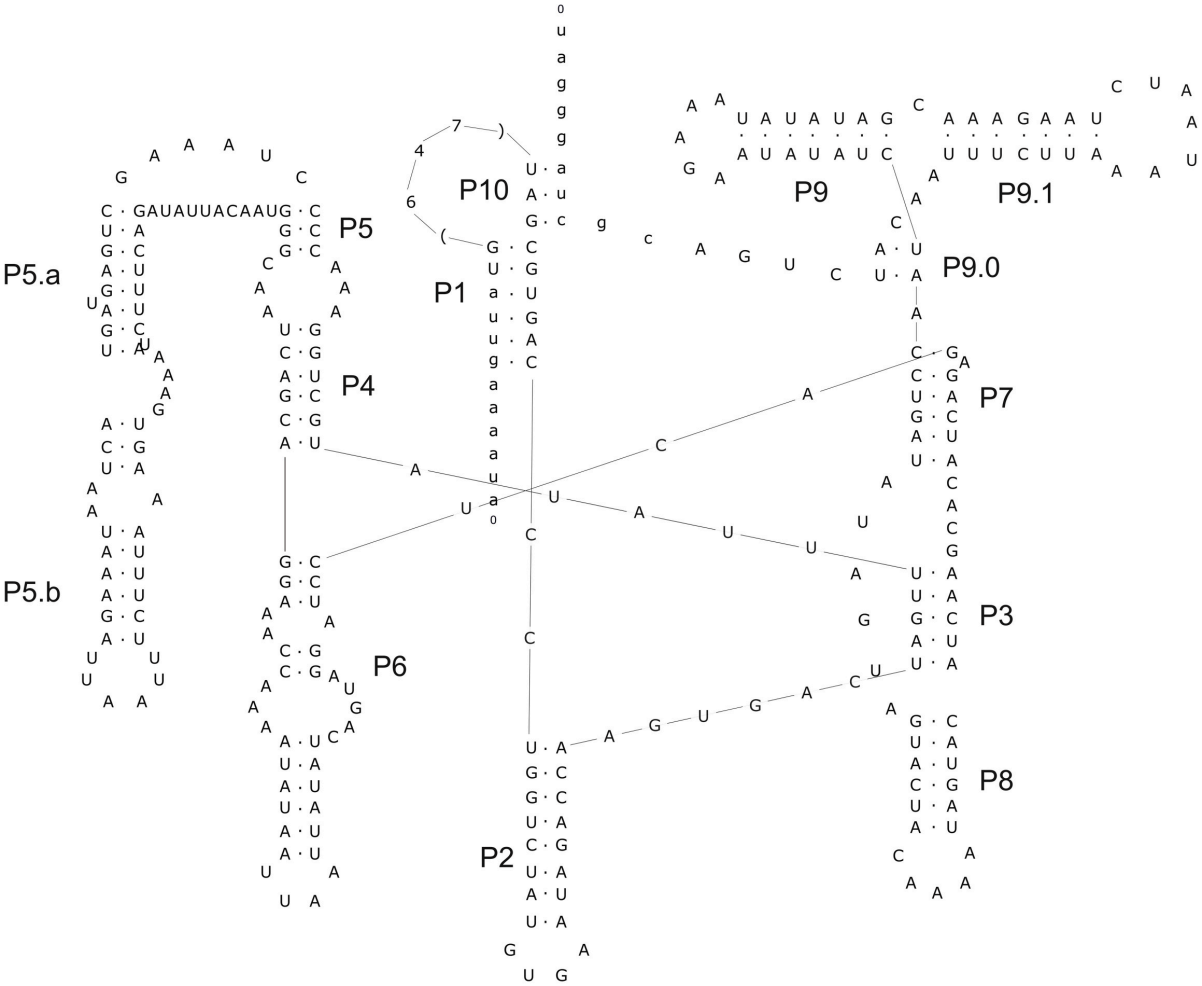
Prediction of secondary structure for intron Cne.mL2439. The analysis was performed with Mfold program online, allowing the identification of major loops and domains. The conserved helices and peripheral elements classify Cne.ml2439 and Cga.mL2439 as typical group-I introns belonging to the IB2 sub-group. The design of the structure was carried out in the Inkscape v.0.91 program. The secondary structure above was drawn using the intron-sequenced WM628 reference strain (FC4 isolate).

**Figure 5:**
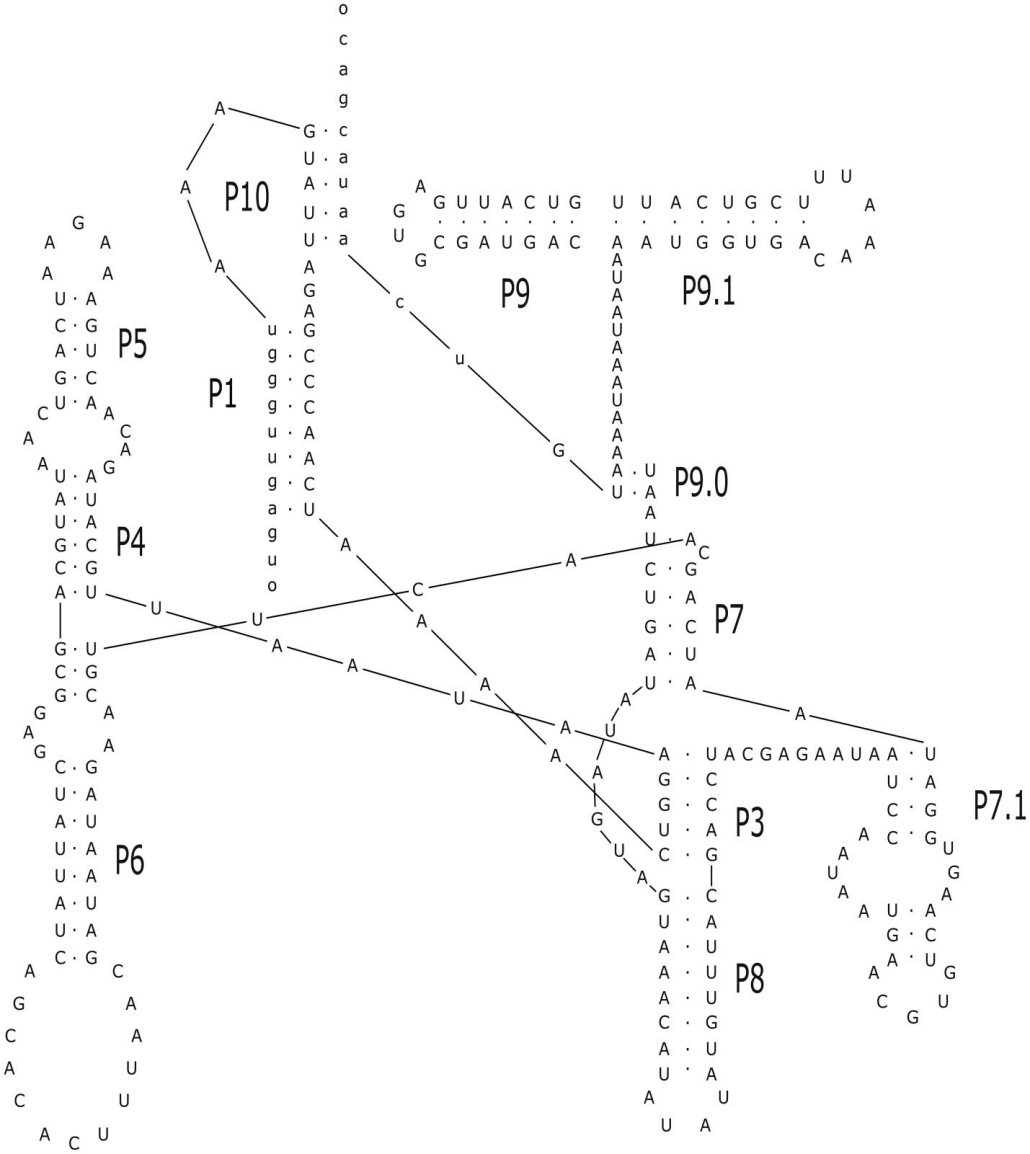
Prediction of secondary structure for the intron Cne.mL2584. The analysis was performed with Mfold program online, allowing the identification of major loops and domains. The conserved helices and peripheral elements classify Cne.ml2584 and Cga.mL2584 as typical group-I introns belonging to the IA1 sub-group. The design of the structure was carried out in the Inkscape v.0.91 program. The secondary structure above was drawn using the intron-sequenced WM629 reference strain (FC2 isolate).

The alignment of introns Cne.mL2439/Cga.mL2439 and Cne.mL2584/Cga.mL2584 to the RF00028 group I intron RFAM model was highly significant for both introns (Supporting Information, Table S2). In addition, several sequence characteristics and the presence of conserved domains were those of archetypal group I introns. Finally, the prediction of their secondary structures (Figures 4, 5) and the analysis of their conserved helical sequences, core domains, junctions and other peripheral elements placed Cne.mL2439/Cga.mL2439 in the IB2 sub-group and Cne.mL2584/Cga.mL2584 in IA1, according to the classification proposed by Michel & Westhof (Michel and Westhof, 1990). Despite the size differences among introns identified here, Cne.mL2439/Cga.mL2439 and Cne.mL2584/Cga.mL2584 showed the same helices and core domains for all isolates in the alignment (slight sequence differences were observed only in non-catalytic helices sites).

### Homing endonuclease genes are responsible for the differences in intron sizes in the mitochondrial LSU rRNA gene of *cryptococcus*

The length polymorphisms related to some molecular types are mainly caused by the presence of HEG. A search in the conserved domain database predicted that some Cne.ml2504 and Cne.mL2449 introns have LAGLIDADG_2 HEG motifs, whereas Cne.mL2439 and Cga.mL2439 introns have conserved motifs for the GIY-YIG HEG family. Cne.mL2584 and Cga.mL2584 do not have HEG motifs (Table 1).

**Table 1:** Presence of homing endonuclease genes (HEGs) in mitochondrial *LSU rRNA* introns from *C. neoformans* and *C. gattii*.

**Intron**	**Length (bp)**	**Homing endonuclease gene**	**Position (bp interval)**	**Genotypes**	**Accession**
Cne.mL2584	250	–	–	VNIII/VNIV	–
Cga.mL2584	252	–	–	VGIII	–
	241	–	–	VGI	–
Cne.mL2504	1,032	LAGLIDADG_2	150–662	VNI	pfam03161
	417	–	–	VNIII/VNIV	–
Cga.mL2504	390	–	–	VGIII	–
	344	–	–	VGI	–
Cne.mL2449	1,169	LAGLIDADG_2	306–830	VNIII/VNIV	pfam03161
	1,168	LAGLIDADG_2	–	VNIV	pfam03161
	579	–	–	VNIII	–
	578	–	–	VNIV	–
Cne.mL2439	1,073	GIY-YIG	610–900	VNII	pfam01541
	925	–	–	VNIII	–
	716	–	–	VNIV	–
Cga.mL2439	1,073	GIY-YIG	617–901	VGIII	pfam01541
	1,059	GIY-YIG	617–907	VGIII/VGIV	pfam01541
	346	–	–	VGI	–

*Search of the Conserved Domain Database indicated the presence of homing endonuclease motifs for the introns studied here. (−) stands for HEG absence*.

### Mitochondrial LSU rRNA group I introns from *C. neoformans* and *C. gattii* are similar to autocatalytic introns in other mitochondrial genes from distinct taxa

Phylogenetic analysis was performed comparing all group I introns studied here with similar sequences in GenBank (Supporting Information, Table S3), using the blastn tool to address the movement and evolution of these mobile elements among different microbial species and genes.

**Figure 6:**
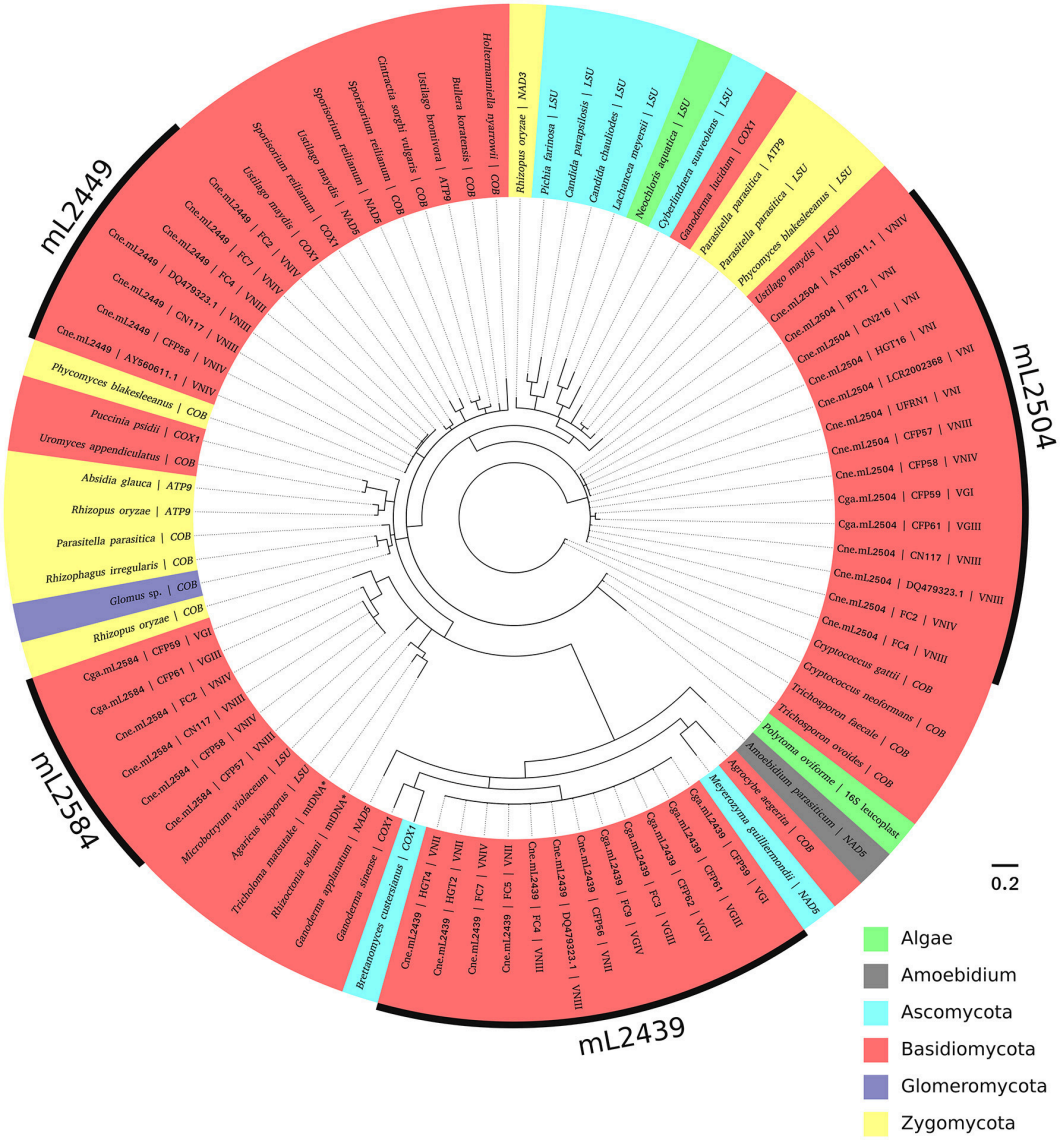
Phylogenetic analysis of the introns and their similar sequences using Bayesian inference. The evolutionary history of the four introns was inferred based on MrBayes under an independent substitution model. Each analysis generated 10,000,000 random trees, with trees and other values saved every 500 generations. All positions containing gaps were deleted. Homing endonuclease regions were not included in the analysis. Evolutionary analyses were conducted in MrBayes (v.3.2.6), viewed using FigTree (v.1.4.2) and edited in Inkscape (v. 0.91). Most of the branches were supported with high posterior probability values (showed in Figure S1).

According to the phylogenetic tree (Figure 6, Supporting Information Figure S1), each of the four introns in the mitochondrial *LSU rRNA* from *Cryptococcus* constitutes a singular clade, close to group I introns in other mitochondrial genes, such as *COX1, COX2, COX3, NAD5, ATP9, COB*, and *LSU* in other fungi and a few non-fungal species, such as *Amoebidium* and algae (*Polytoma oviforme* and *Neochloris aquatica*). Therefore, introns at one site in the *LSU rRNA* gene are not invading other sites in the same gene. The elements at the 2504 position form a basal non-monophyletic clade with no apparent divergence between the Cne.mL2504 and Cga.mL2504 introns. While Cga.mL2504 constitute a monophyletic clade, Cne.mL2504 is paraphyletic (it should include Cga.mL2504 introns to be monophyletic), which could indicate that the intron at the 2504 position has been horizontally transferred between species. Introns at the 2449, 2584, and 2439 positions constitute distinct, monophyletic and well-defined clades. Introns at the 2449 site occur only in *C. neoformans* and are closely related to other introns from other mitochondrial genes, mostly from other basidiomycetes. The 2584 introns also form a monophyletic clade, closer to *LSU rRNA* introns from other basidiomycetes; however, Cga.mL2584 is paraphyletic because it shares a common ancestor with Cne.mL2584, which is another possible indication of horizontal transfer of these elements between *C. neoformans* and *C. gattii*. Cne.mL2439 and Cga.mL2439 are together a monophyletic group, closely related to *COX1* introns from an ascomycete and a basidiomycete, with no informative polymorphism to distinguish *Cryptococcus* species.

### Intron absence in the mitochondrial LSU rRNA in cryptococcus is associated with the most virulent genotypes

We detected an association between intronless isolates and those genotypes shown in some reports to be the most virulent and less susceptible to antifungal agents (VGI, VGII, VNI, and VNIV) (Chong et al., 2010; Hagen et al., 2010; Iqbal et al., 2010; Trilles et al., 2012). As Table 2 shows, Cne.mL2584/Cga.mL2584, Cne.mL2449, and Cne.mL2439/Cga.mL2439 introns were associated with those genotypes shown to be less virulent (VGIII, VGIV, VNII, and VNIII). Of note, Cne.mL2504/Cga.mL2504 had a borderline *p*-value of 0.063. The overall analysis considered the presence of at least one intron, regardless intron quantity and site insertion in *LSU rRNA* for each isolate, and it was observed that intron presence is associated with those genotypes reported to be less virulent (*p* < 0.001). Future work involving virulence assays will be needed to confirm this association between intron presence and low virulence.

**Table 2:** Intron presence is over-represented in non-virulent genotypes.

**Intron**		**Genotypes**		***p*-value**
		Non-virulent^*^	**Virulent^#^**	
Cne.mL2584/	Absent	8 (72.73%)	63 (95.45%)	0.035
Cga.mL2584	Present	3 (27.27%)	3 (4.55%)	
Cne.mL2504/	Absent	7 (63.64%)	58 (87.88%)	0.063
Cga.mL2504	Present	4 (36.36%)	8 (12.12%)	
Cne.mL2449	Absent	8 (72.73%)	63 (95.45%)	0.035
	Present	3 (27.27%)	3 (4.55%)	
Cne.mL2439/	Absent	1 (9.09%)	64 (96.97%)	< 0.001
Cga.mL2439	Present	10 (90.91%)	2 (3.03%)	
Introns overall	Absent	0 (0.00%)	57 (86.36%)	< 0.001
	Present	11 (100.00%)	9 (13.64%)	

*Intron column indicates presence of at least one intron (i.e., Present) or lack of all introns (i.e., Absent)*.

* *Genotypes reported to be less virulent and susceptible to antifungals agents (VNII, VNIII, VGIII, VGIV) according to references (Chong et al., 2010; Hagen et al., 2010; Iqbal et al., 2010; Trilles et al., 2012)*.

# *Genotypes reported as more virulent and susceptible to antifungals agents (VNI, VGI, VGII, VNIV) according to references (Chong et al., 2010; Hagen et al., 2010; Iqbal et al., 2010; Trilles et al., 2012)*.

*P-values obtained from Fisher exact tests*.

### The presence of introns influences the fungal susceptibility to amphotericin B and 5-fluorocytosine

MIC values obtained for the antifungals amphotericin B, 5-fluorocytosine and itraconazole are presented in Supporting Information, Table S4. The ordered logistic regression models estimate the odds of being in a higher MIC based on the values of predictor variables (i.e., introns and species). The magnitude and direction of the effect are given by the beta coefficients (i.e., a positive beta represents an increase in the odds of being in a higher MIC category, whereas a negative beta means decreased odds of being in a higher MIC, both in natural log scale). In other words, the beta value reflects the effect of introns and species on fungal susceptibility to antifungal drugs.

Table 3 summarizes the results of regression analyses. The best models (i.e., lowest AIC value) for amphotericin B and itraconazole were the interaction and simple models, respectively. For 5-fluorocytosine, the two models were equivalent, so we chose the interaction model since it provided the lowest *p*-value (*p* = 0.026) for the beta coefficient estimate of the intron term. Therefore, based on these selected models, the results indicate: (1) the presence of introns is associated with a higher amphotericin B MIC (*p* = 0.010); (2) *C. neoformans* species is associated with a higher amphotericin B MIC (*p* = 0.016); (3) *C. neoformans* is associated with a lower itraconazole MIC (*p* = 0.036); and (4) the presence of introns is associated with a lower 5-fluorocytosine MIC (*p* = 0.026).

**Table 3:** Ordered logistic models used to estimate the effect of intron and species on MIC.

**Model**	**Response variable**	**Predictor variables**	**Beta Coeff**.	**Standard error**	***p*-value**	**AIC**
Simple	Amphotericin B MIC	Introns	0.5337	0.6200	0.389	97.2458
		Species	0.5543	0.6321	0.381	
Interaction	Amphotericin B MIC	Introns	2.9354	1.1347	**0.010**	91.5226
		Species	2.3037	0.9553	**0.016**	
		introns*species	−3.7698	1.4362	**0.009**	
Simple	Itraconazole MIC	Introns	0.3705	0.8565	0.665	66.8341
		Species	−1.8186	0.8690	**0.036**	
Interaction	Itraconazole MIC	Introns	0.4407	1.1294	0.696	68.8251
		Species	−1.7354	1.2224	0.156	
		introns*species	−0.1633	1.7179	0.924	
Simple	5-fluorocytosine MIC	Introns	−1.2032	0.6361	0.059	113.5133
		Species	0.8923	0.6352	0.160	
Interaction	5-fluorocytosine MIC	Introns	−2.3165	1.0395	**0.026**	113.5577
		Species	0.0163	0.8892	0.985	
		introns*species	1.7803	1.2894	0.167	

*Regression models were implemented with introns coded as: presence of at least one intron = 1 and lack of all introns = 0 (reference group); and species coded as: C. neoformans = 1 and C. gattii = 0 (reference group). Each model analyzed a total number of 40 isolates: 13 VNI, 4 VNII, 3 VNIII, 5 VNIV, 2 VGI, 9 VGII, 2 VGIII, and 2 VGIV. p-values in bold are statistically significant*.

**Figure 7:**
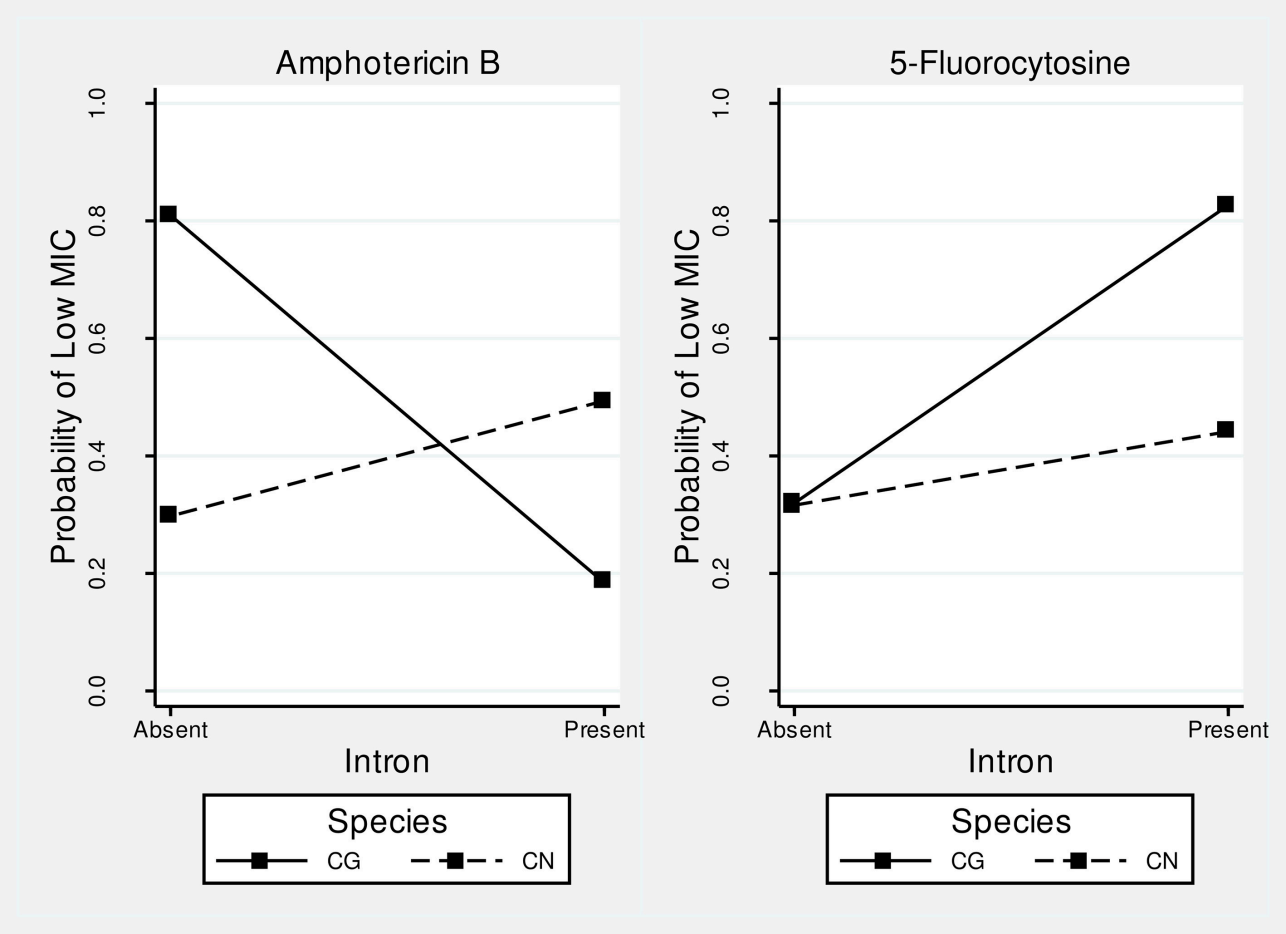
Interaction plots for amphotericin B and 5-fluorocytosine. The higher the probability of a low MIC, the more susceptible is the fungus. The differences in slopes suggest that the intron effect differs in the two species. For amphotericin B, the presence of introns leads to resistance in *C. gattii* but susceptibility in *C. neoformans*. For 5-fluorocytosine, the intron presence leads to susceptibility in both species, although the magnitude of the effect is greater in *C. gattii*. Overall, the effect of introns seems to be more pronounced in *C. gattii* (i.e., higher slopes).

Besides these marginal effects, the species clearly modifies the effect of intron presence on the amphotericin B MIC (*p* = 0.009 for the interaction term). For example, in *C. gattii* intron presence is more associated with higher MIC values, whereas in *C. neoformans* it is associated with lower MIC values (Figure 7), showing an opposite effect of intron presence in the different species. The effect of intron presence on the 5-fluorocytosine MIC also seems to be modified by species. The presence of a group I intron in *C. gattii* is associated with lower MIC values than in *C. neoformans* (Figure 7); however, the *p*-value for the interaction term was not significant (*p* = 0.167).

## Discussion

Group I introns are mobile autocatalytic elements that have invaded tRNA, rRNA and protein coding genes throughout evolution. Their distribution is diverse since they can be found in bacterial, phage, viral and organelle genomes and often in nuclear rRNA genes (Haugen et al., 2005; Hausner et al., 2014). Since these elements are polymorphic in their presence and sequence, they are strong candidates for molecular markers. For this reason, we studied the potential of group I introns in the mitochondrial *LSU rRNA* of *C. neoformans* and *C. gattii* as a drug susceptibility indicator since in other fungi, their presence is related to attenuated virulence and antifungal susceptibility (Mercure et al., 1993; Miletti and Leibowitz, 2000; Jayaguru and Raghunathan, 2007).

An important finding of this work was the identification of two new introns in the mitochondrial *LSU rRNA* gene whose sequences and structures have not been previously described. These introns are present in both species (*C. neoformans* and *C. gattii)* and were named Cne.mL2439 and Cne.mL2584 in *C. neoformans* and Cga.mL2439 and Cga.mL2584 in *C. gattii*. Their predicted secondary structures show the presence of the main helical domains and consensus peripheral elements typical of group I introns, such as the substrate-binding domain (P1 and P10 helices), the P4/P6 scaffolding domain (P4, P5, and P6 helices) and the P3/P9 catalytic domain (P3, P7, P8, and P9) that contains the guanosine-5′-triphosphate (GTP) binding site within the P7 helix (Michel and Westhof, 1990; Cech et al., 1994; Li and Zhang, 2005), indicating full functionality of these elements.

HEGs are responsible for the length variation of the introns studied here. We observed that these elements may be present or absent in some introns (Cne.mL2449 and Cne.mL2504), suggesting that some group I introns remain mobile in the genome by the homing process. HEGs are DNA-cleaving enzymes that recognize long-specific DNA sequences and performs a double-strand break (DSB), promoting the specific duplication of group I introns (and other self-splicing intervening sequences, such as group II introns and inteins). After introduction of the DSB in the intronless allele, homologous recombination uses the intron-containing allele as a template for repair (Stoddard, 2011). However, this is not the only way for group I introns to move. Another known mechanism is reverse splicing. In this process, intron mobility initiates when the excised intron attacks and reinserts itself into the ligated RNA exons guided by a short internal sequence of 4–6 nucleotides, and then integrates into genome after its reverse transcription (Birgisdottir and Johansen, 2005; Hedberg and Johansen, 2013). Reverse splicing is a less specific process and, for this reason is considered a possible mechanism for explaining the spread of these self-splicing elements into new and heterologous sites (Bhattacharya et al., 2005).

In the phylogeny (Figure 6), we observed that sequences of each intron (Cne.mL2439/Cga.mL2439, Cne.mL2449, Cne.mL2504, and Cne.mL2584/Cga.mL2584) were grouped separately, arguing for a distinct evolutionary history of the *LSU rRNA* group I introns in *C. neoformans* and *C. gattii*. This result shows, therefore, that allelic introns in the mitochondrial *LSU rRNA* genes of *C. neoformans* and *C. gattii* share more similarity among themselves than with non-allelic introns of the same gene, which supports the interpretation that simultaneous invasions at different sites of *LSU rRNA* by the same intron are not occurring.

Furthermore, phylogenetic analysis revealed a similarity of the introns studied here with other mitochondrial autocatalytic introns from distinct genes in different fungal phyla, as well as in non-fungal taxa, such as algae (*16S* gene, leucoplast genome, and *LSU* gene, mitochondrial genome) and *Amoebidium* (*NAD5* gene, mitochondrial genome). This could indicate a possible horizontal transfer. In fact, autocatalytic introns are very ancient and frequently transferred laterally, so that the main hypothesis for their sporadic distribution considers their presence in genomes even before the endosymbiosis event by prokaryotes, which would eventually become intracellular organelles in eukaryotic cells. Indeed the structural similarity between some autocatalytic introns (group II introns) and spliceosomal introns give us some clues about the origin of interrupted genes in eukaryotic nuclear genomes (Koonin, 2006; Rogozin et al., 2012). An ancient invasion of group I introns might explain their presence in so many organelles and even in nuclear genes of some eukaryotes (in *rRNA* genes, exclusively) (Hedberg and Johansen, 2013). Therefore, the similarity of group I introns among fungi and other taxa observed here could be explained by old invasions (pre-dating the endosymbiosis event) and by the combination of two main mobility mechanisms, i.e., reverse splicing that results in intron occupation of heterologous sites, and action of HEGs responsible for the fast spread and fixation of the group I intron in a population (Belfort and Perlman, 1995; Haugen et al., 2004; Birgisdottir and Johansen, 2005; Hedberg and Johansen, 2013). Besides reverse splicing, HEGs could also themselves be responsible for heterologous invasions under particular physiological conditions. For instance, the exposure of I-TevI (the endonuclease from the thymidylate synthase gene of T4 phage) to oxidative stress disrupts its fidelity, making the invasion of heterologous sites easier (Robbins et al., 2011). Taking into account the fact that the mitochondrial environment is constantly under this kind of stress, we could hypothesize that HEGs are more “promiscuous” in this organelle, increasing the overall number of group I introns in its genome.

Our results showed that the presence/absence of group I introns within the same genotype can vary, as observed for VNI, VNIV, and VGI. Similarly, HEGs may also be present or absent in Cne.mL2449 and Cne.mL2504 introns in some molecular types. These findings are congruent with a homing cycle mechanism, which suggests that HEGs are continually gained, fixed, degenerated, and lost in a cyclical manner. After an intron-HEG becomes fixed in a population there is no selective pressure for HEG functionality, so this domain may accumulate degenerative mutations, become inactivated and eventually lost, explaining their absence in some introns and also the intron absence itself because once the HEG is no longer functional, there is no intron mobility into empty alleles (Goddard and Burt, 1999; Haugen et al., 2005).

All the isolates evaluated here that belong to the VGII genotype, considered the most resistant genotype to antifungals, especially to azoles (Trilles et al., 2012), do not have an intron in the mitochondrial *LSU rRNA* gene. It is worth noting that VGII has become an important genotype within *C. gattii* since its emergence in a Vancouver (Canada) outbreak with novel, highly virulent strains/subtypes (VGIIa, VGIIb, and VGIIc) that dispersed into neighboring regions including the United States (Kidd et al., 2004; Byrnes et al., 2010). This high virulence profile is associated with its capacity to produce fused and tubular mitochondria, probably due to the up-regulation of some nuclear-encoded proteins with important functions for this organelle. It was hypothesized that this unusual mitochondrial morphology could be a protective response of the pathogen against the intracellular stress (inside macrophages) (Ma et al., 2009; Ma and May, 2010). Similarly, the VNI genotype, known as the *C. neoformans* molecular type less susceptible to antifungal agents and the major cause of mortality among *C. neoformans* genotypes (Trilles et al., 2012), does not have introns at positions 2439, 2449, and 2584. In this report, we observed the absence of introns in most VNI strains (87.2%), indicating, together with intron absence in VGII, the possible association of these elements with virulence. Since our study was based only on a sequencing approach and literature information on the pathogenic profiles of different *Cryptococcus* genotypes, this association will need to be further investigated by experimental virulence assays.

The presence of introns in such an important gene requires a perfect splicing under any physiological condition. Despite no existing report on splicing inhibition of group I introns from *Cryptococcus*, studies of the splicing kinetics for other introns gives us some clues about possible interfering conditions. For instance, nicotinamide coenzymes, such as NADP^+^ and NADPH^+^, are known to inhibit the self-splicing of the group I intron present in the thymidylate synthase gene of T4 phage (Park and Kim, 2001; Kim and Park, 2003). Those coenzymes are largely present in the mitochondrial environment and we hypothesize they could decrease the efficiency of mitochondrial introns splicing and therefore the fungal virulence, which could explain the possible association between intron presence and low virulence genotypes. Experimental data associating virulence profiles for *Cryptococcus* isolates with and without introns in their mitochondrial genome are necessary to confirm this hypothesis.

Reinforcing these previous observations, we also found a strong association between intronless strains and high MIC values for the 5-fluorocytosine drug, which is corroborated by other studies in *C. albicans*. This antifungal drug disrupts self-splicing of the Ca.*LSU rRNA* group I intron in this opportunistic yeast by the insertion of 5-fluorouracil residues into the *rRNA* (Mercure et al., 1993, 1997). Once the intron is retained, the resulting rRNA is nonfunctional and, therefore, incompatible with cell survival. Here, we hypothesize that something similar may also occur for 5-fluorocytosine in isolates of *C. neoformans* and *C. gattii*, which have autocatalytic group I introns in *LSU rRNA*. Experimental *in vitro* assays will need to be conducted to understand the exact effect of 5-fluorocytosine on the self-splicing of the group I introns in *Cryptococcus* spp.

Interestingly, and divergently from the observed effect of 5-fluorocytosine, high MIC values for amphotericin B were associated with intron presence in *C. gattii* (opposite to that observed for *C. neoformans*). In fact, since the mechanism of action of this drug focuses on the fungal cell membrane, altering its permeability, and not on nucleic acid metabolism as does 5-fluorocytosine, we expected no association between MIC and intron presence, as seen for the itraconazole drug. This may simply suggest that the presence of these introns in some isolates might be in linkage to other genetic markers responsible for these increased MIC values. However, again this is an area for future research on more isolates of both species with specific antifungal tests, as well as *in vitro* assays on the action of these drugs on intron splicing efficiency.

Concerning the association between species and MIC values for the three drugs tested here, according to the statistical analysis, the *C. neoformans* evaluated here showed higher MIC values for amphotericin B than did *C. gattii*, which could indicate a peculiarity of these *C. neoformans* isolates (most from VNI genotype) from Rio Grande do Norte, Brazil, since usually no significant difference is observed for this drug, as previously reported (Trilles et al., 2012). Oppositely, the *C. neoformans* isolates evaluated here were more susceptible to itraconazole than *C. gattii* (*p* = 0.036), which could reflect the already observed difference of susceptibility between VNI and VGII (Trilles et al., 2012). In the case of 5-fluorocytosine, there was no significant difference of MIC values between *Cryptococcus* species complex, suggesting that intron presence (*p* = 0.026), rather than species, is actually the relevant factor for the increased susceptibility to this drug.

In this study, most of the *Cryptococcus* isolates belonged to VNI and VGII genotypes, which are the most frequent ones in Brazil (Santos et al., 2008; Matos et al., 2012; Favalessa et al., 2014). Thus, collaboration with other reference centers in medical mycology will be necessary for a global investigation of the overall potential of mitochondrial group I introns in *LSU rRNA* genes, and in other mitochondrial genes, as molecular markers for all genotypes of *C. neoformans* and *C. gattii*, given the polymorphic characteristic of these elements. Nevertheless, this was the first study in which mitochondrial autocatalytic introns of the *LSU rRNA* gene from *C. neoformans* and *C. gattii* were investigated in a significant number of isolates from different genotypes. Our main findings point to a large polymorphism in group I introns, including the secondary structure prediction of two new introns, as well as to a variation in their presence and nucleotide lengths among the different genotypes of *Cryptococcus* not previously described. Despite the fact that these introns cannot differentiate completely all the *Cryptococcus* genotypes, an association was observed between intron absence and the genotypes known as most virulent, such as VGII, VGI, VNI, and VNIV. Moreover, our antifungal susceptibility assay revealed a suggestive relationship between intron presence and susceptibility to 5-fluorocytosine, indicating that these elements might be therapeutic targets, as previously mentioned for *C. albicans* (Mercure et al., 1993, 1997). Further *in vitro* studies will address the effect of these and other drugs on group I intron splicing in the pathogenic *Cryptococcus* species.

## Conclusion

*Cryptococcus neoformans* and *C. gattii* are important worldwide-distributed pathogens that cause systemic mycoses with prolonged treatment and in some cases, depending on their genotype, present a resistance profile to antifungal drugs. Genotype-specific diagnosis using molecular markers for drug susceptibility and virulence may contribute to a correct treatment, improving patient prognosis. Mitochondrial group I introns in *LSU rRNA* gene from *Cryptococcus* are good candidates for such markers, since our results showed that their absence seems to be associated with those genotypes shown to be the most virulent and to have high MIC values for 5-fluorocytosine, corroborating their potential as new and alternative drug targets. In addition, the discovery of two new introns in mitochondrial *LSU rRNA* gene shows that the polymorphism of these elements in mtDNA may be even higher than initially thought. Since they are present in extremely important genes for fungal cell survival, mainly during host-pathogen interactions, evolutionary studies on their distribution dynamics in the mitochondrial genome of *C. neoformans* and *C. gattii* might reveal new markers for virulence and drug susceptibility.

## Author contributions

FG did the literature review and most experimental procedures and wrote the manuscript, TA, JF, and HR assisted in phylogenetic, infernal/RFAM and intron structure analysis and co-wrote the manuscript, LF statistically analyzed the results and co-wrote the manuscript, SB, MO, and GD supplied most of fungal culture as well as infrastructure for culturing and DNA extractions of *Cryptococcus* and co-wrote the manuscript, RT designed and supervised the experimental procedures as well as organized the writing manuscript.

### Conflict of interest statement

The authors declare that the research was conducted in the absence of any commercial or financial relationships that could be construed as a potential conflict of interest.

## References

[B1] Altschul S. Gish W. Miller W. Myers E. Lipman D. (1990). Basic local alignment search tool. J. Mol. Biol. 215, 403–410. 10.1016/S0022-2836(05)80360-2 2231712

[B2] Belfort M. Perlman P. S. (1995). Mechanisms of intron mobility. J. Biol. Chem. 270, 30237–30240. 10.1074/jbc.270.51.30237 8530436

[B3] Belfort M. Roberts R. J. (1997). Homing endonucleases: keeping the house in order. Nucleic Acids Res. 25, 3379–3388. 10.1093/nar/25.17.3379 9254693 PMC146926

[B4] Bhattacharya D. Reeb V. Simon D. M. Lutzoni F. (2005). Phylogenetic analyses suggest reverse splicing spread of group I introns in fungal ribosomal DNA. BMC Evol. Biol. 5:68. 10.1186/1471-2148-5-68 16300679 PMC1299323

[B5] Birgisdottir Å. B. Johansen S. (2005). Site-specific reverse splicing of a HEG-containing group I intron in ribosomal RNA. Nucleic Acids Res. 33, 2042–2051. 10.1093/nar/gki341 15817568 PMC1074745

[B6] Boekhout T. Theelen B. Diaz M. Fell J. W. Hop W. C. J. Abeln E. C. A.. (2001). Hybrid genotypes in the pathogenic yeast *Cryptococcus neoformans*. Microbiology 147, 891–907. 10.1099/00221287-147-4-891 11283285

[B7] Brandt M. E. Hutwagner L. C. Kuykendall R. J. Pinner R. W. (1995). Comparison of multilocus enzyme electrophoresis and random amplified polymorphic DNA analysis for molecular subtyping of *Cryptococcus neoformans*. the cryplococcal disease active surveillance group. J. Clin. Microbiol. 33, 1890–1895. 7665665 10.1128/jcm.33.7.1890-1895.1995 PMC228292

[B8] Byrnes E. J. Li W. Lewit Y. Ma H. Voelz K. Ren P.. (2010). Emergence and pathogenicity of highly virulent *Cryptococcus gattii* genotypes in the northwest United States. PLoS Pathog. 6:e1000850. 10.1371/journal.ppat.1000850 20421942 PMC2858702

[B9] CLSI-Clinical Laboratory Standards Institute (2008a). Reference Method for Broth Dilution Antifungal Susceptibility Testing of Yeasts: Approved Standard, 3rd Edn. CLSI Document M27–A3. Wayne, PA: Clinical Laboratory Standards Institute.

[B10] CLSI-Clinical Laboratory Standards Institute (2008b). Reference Method for Broth Dilution Antifungal Susceptibility Testing of Yeasts; Fourth Informational Supplement. CLSI Document M27–S4. Wayne, PA: Clinical Laboratory Standards Institute.

[B11] Cannone J. J. Subramanian S. Schnare M. N. Collett J. R. D'Souza L. M. Du Y.. (2002). The comparative RNA web (CRW) site: an online database of comparative sequence and structure information for ribosomal, intron, and other RNAs. BMC Bioinformatics 3:2. 10.1186/1471-2105-3-2 11869452 PMC65690

[B12] Cech T. R. Damberger S. H. Gutell R. R. (1994). Representation of the secondary and tertiary structure of group I introns. Nat. Struct. Biol. 1, 273–280. 10.1038/nsb0594-273 7545072

[B13] Chen S. C. A. Meyer W. Sorrell T. C. (2014). *Cryptococcus gattii* infections. Clin. Microbiol. Rev. 27, 980–1024. 10.1128/CMR.00126-13 25278580 PMC4187630

[B14] Chong H. S. Dagg R. Malik R. Chen S. Carter D. (2010). *In vitro* susceptibility of the yeast pathogen cryptococcus to fluconazole and other azoles varies with molecular genotype. J. Clin. Microbiol. 48, 4115–4120. 10.1128/J.C.M.01271-10 20844209 PMC3020851

[B15] Cowen L. E. Sanglard D. Howard S. J. Rogers P. D. Perlin D. S. (2014). Mechanisms of antifungal drug resistance. Cold Spring Harb. Perspect. Med. 5:a019752. 10.1101/cshperspect.a019752 25384768 PMC4484955

[B16] Dismukes W. E. (2000). Introduction to Antifungal Drugs. Clin. Infect. Dis. 30, 653–657. 10.1086/313748 10770726

[B17] Disney M. D. Matray T. Gryaznov S. M. Turner D. H. (2001). Binding enhancement by tertiary interactions and suicide inhibition of a *Candida albicans* group I intron by phosphoramidate and 2'-O-methyl hexanucleotides. Biochemistry 40, 6520–6526. 10.1021/bi002009j 11371215

[B18] D'Souza C. A. Kronstad J. W. Taylor G. Warren R. Yuen M. Hu G. (2011). Genome variation in *Cryptococcus gattii*, an emerging pathogen of immunocompetent hosts. MBio 2:e00342-10. 10.1128/mBio.00342-10 21304167 PMC3037005

[B19] Farrer R. A. Desjardins C. A. Sakthikumar S. Gujja S. Saif S. Zeng Q.. (2015). Genome evolution and innovation across the four major lineages of *Cryptococcus gattii*. 6, 1–12. 10.1128/mBio.00868-15 26330512 PMC4556806

[B20] Favalessa O. C. de Paula D. A. J. Dutra V. Nakazato L. Tadano T. Lazera M.. (2014). Molecular typing and *in vitro* antifungal susceptibility of Cryptococcus spp from patients in midwest Brazil. J. Infect. Dev. Ctries 8, 1037–1043. 10.3855/jidc.4446 25116671

[B21] Goddard M. R. Burt A. (1999). Recurrent invasion and extinction of a selfish gene. Proc. Natl. Acad. Sci. U.S.A. 96, 13880–13885. 10.1073/pnas.96.24.13880 10570167 PMC24159

[B22] Gullo F. P. Rossi S. A. Sardi J. D. C. O. Teodoro V. L. I. Mendes-Giannini M. J. S. Fusco-Almeida A. M. (2013). Cryptococcosis: epidemiology, fungal resistance, and new alternatives for treatment. Eur. J. Clin. Microbiol. Infect. Dis. 32, 1377–1391. 10.1007/s10096-013-1915-8 24141976

[B23] Hagen F. Illnait-Zaragozi M. T. Bartlett K. H. Swinne D. Geertsen E. Klaassen C. H. W.. (2010). *In vitro* antifungal susceptibilities and amplified fragment length polymorphism genotyping of a worldwide collection of 350 clinical, veterinary, and environmental *Cryptococcus gattii* isolates. Antimicrob. Agents Chemother. 54, 5139–5145. 10.1128/AAC.00746-10 20855729 PMC2981230

[B24] Hagen F. Khayhan K. Theelen B. Kolecka A. Polacheck I. Sionov E.. (2015). Recognition of seven species in the *Cryptococcus gattii*/ *Cryptococcus neoformans* species complex. Fungal Genet. Biol. 78, 16–48. 10.1016/j.fgb.2015.02.009 25721988

[B25] Haugen P. Runge H. J. Bhattacharya D. (2004). Long-term evolution of the S788 fungal nuclear small subunit rRNA group I introns long-term evolution of the S788 fungal nuclear small subunit rRNA group I introns. RNA 10, 1084–1096. 10.1261/rna.5202704 15208444 PMC1370599

[B26] Haugen P. Simon D. M. Bhattacharya D. (2005). The natural history of group I introns. Trends Genet. 21, 111–119. 10.1016/j.tig.2004.12.007 15661357

[B27] Hausner G. Hafez M. Edgell D. R. (2014). Bacterial group I introns: mobile RNA catalysts. Mob. DNA 5:8. 10.1186/1759-8753-5-8 24612670 PMC3984707

[B28] Hedberg A. Johansen S. D. (2013). Nuclear group I introns in self-splicing and beyond. Mob. DNA 4:17. 10.1186/1759-8753-4-17 23738941 PMC3679873

[B29] Iqbal N. DeBess E. E. Wohrle R. Sun B. Nett R. J. Ahlquist A. M.. (2010). Correlation of genotype and *in vitro* susceptibilities of *Cryptococcus gattii* strains from the pacific northwest of the United States. J. Clin. Microbiol. 48, 539–544. 10.1128/JCM.01505-09 20007380 PMC2815610

[B30] Jayaguru P. Raghunathan M. (2007). Group I intron renders differential susceptibility of *Candida albicans* to Bleomycin. Mol. Biol. Rep. 34, 11–17. 10.1007/s11033-006-9002-1 17115251

[B31] Johansen S. Haugen P. (2001). A new nomenclature of group I introns in ribosomal DNA. RNA 7, 935–936. 10.1017/S1355838201010500 11453066 PMC1370146

[B32] Kidd S. E. Hagen F. Tscharke R. L. Huynh M. Bartlett K. H. Fyfe M.. (2004). A rare genotype of *Cryptococcus gattii* caused the cryptococcosis outbreak on Vancouver Island (British Columbia, Canada). Proc. Natl. Acad. Sci. U. S. A. 101, 17258–17263. 10.1073/pnas.0402981101 15572442 PMC535360

[B33] Kim J. H. Park I. K. (2003). Inhibition of the group I ribozyme splicing by *NADP*+. Mol. Cell Biochem. 252, 285–293. 14577604 10.1023/a:1025561706522

[B34] Koonin E. V. (2006). The origin of introns and their role in eukaryogenesis: a compromise solution to the introns-early versus introns-late debate? Biol. Direct 1:22. 10.1186/1745-6150-1-22 16907971 PMC1570339

[B35] Kwon-chung K. J. Fraser J. A. Doering T. L. Wang Z. A. Janbon G. Idnurm A.. (2014). Cryptococcus neoformans and *Cryptococcus gattii*, the etiologic agents of cryptococcosis. Cold Spring Harb. Perspect. Med. 4:a019760. 10.1101/cshperspect.a019760 24985132 PMC4066639

[B36] Lambowitz A. Caprara M. (1999). Group I and Group II Ribozymes as RNPs: Clues to the Past and Guides to the Future. Available online at: http://rna.cshl.edu/content/free/chapters/18_rna_world_2nd.pdf.

[B37] Laniado-Laborín R. Cabrales-Vargas M. N. (2009). Amphotericin B: side effects and toxicity. Rev. Iberoam. Micol. 26, 223–227. 10.1016/j.riam.2009.06.003 19836985

[B38] Leal A. L. Faganello J. Bassanesi M. C. Vainstein M. H. (2008). Cryptococcus species identification by multiplex PCR. Med. Mycol. 46, 377–383. 10.1080/13693780701824429 18415847

[B39] Li Z. Zhang Y. (2005). Predicting the secondary structures and tertiary interactions of 211 group I introns in IE subgroup. Nucleic Acids Res. 33, 2118–2128. 10.1093/nar/gki517 15843683 PMC1083426

[B40] Litter J. Keszthelyi A. Hamari Z. Pfeiffer I. Kucsera J. (2005). Differences in mitochondrial genome organization of *Cryptococcus neoformans* strains. Antonie van Leeuwenhoek, Int. J. Gen. Mol. Microbiol. 88, 249–255. 10.1007/s10482-005-8544-x 16284931

[B41] Löytynoja A. Goldman N. (2010). webPRANK: a phylogeny-aware multiple sequence aligner with interactive alignment browser. BMC Bioinformatics 11:579. 10.1186/1471-2105-11-579 21110866 PMC3009689

[B42] Ma H. May R. C. (2010). outbreak on Vancouver Island. J. Infect. Dis. 197–201. 10.1111/j.1469-0691.2010.03222.x PMC3073246 21178442

[B43] Ma H. Hagen F. Stekel D. J. Johnston S. A. Sionov E. Falk R.. (2009). The fatal fungal outbreak on Vancouver Island is characterized by enhanced intracellular parasitism driven by mitochondrial regulation. Proc. Natl. Acad. Sci. U.S.A. 106, 12980–12985. 10.1073/pnas.0902963106 19651610 PMC2722359

[B44] Marchler-Bauer A. Derbyshire M. K. Gonzales N. R. Lu S. Chitsaz F. Geer L. Y.. (2015). CDD: NCBI's conserved domain database. Nucleic Acids Res. 43, D222–D226. 10.1093/nar/gku1221 25414356 PMC4383992

[B45] Matos C. S. De Souza Andrade A. Oliveira N. S. Barros T. F. (2012). Microbiological characteristics of clinical isolates of Cryptococcus spp. in Bahia, Brazil: molecular types and antifungal susceptibilities. Eur. J. Clin. Microbiol. Infect. Dis. 31, 1647–1652. 10.1007/s10096-011-1488-3 22278291 PMC3364408

[B46] Mercure S. Cousineau L. Montplaisir S. Belhumeur P. Lemay G. (1997). Expression of a reporter gene interrupted by the *Candida albicans* group I intron is inhibited by base analogs. Nucleic Acids Res. 25, 431–437. 10.1093/nar/25.2.431 9016575 PMC146449

[B47] Mercure S. Montplaisir S. Lemay G. (1993). Correlation between the presence of a self-splicing intron in the 25S rDNA of C.albicans and strains susceptibility to 5-fluorocytosine. Nucleic Acids Res. 21, 6020–6027. 10.1093/nar/21.25.6020 7904747 PMC310489

[B48] Meyer W. Aanensen D. M. Boekhout T. Cogliati M. Diaz M. R. Esposto M. C.. (2009). Consensus multi-locus sequence typing scheme for *Cryptococcus neoformans* and *Cryptococcus gattii*. Med. Mycol. 47, 561–570. 10.1080/13693780902953886 19462334 PMC2884100

[B49] Meyer W. Casta-eda A. Jackson S. Huynh M. Casta-eda E. Arechavala A.. (2003). Molecular typing of IberoAmerican *Cryptococcus neoformans* isolates. Emerg. Infect. Dis. 9, 189–195. 10.3201/eid0902.020246 12603989 PMC2901947

[B50] Michel F. Westhof E. (1990). Modelling of the three-dimensional architecture of group I catalytic introns based on comparative sequence analysis. J. Mol. Biol. 216, 585–610. 10.1016/0022-2836(90)90386-Z 2258934

[B51] Miletti K. E. Leibowitz M. J. (2000). Pentamidine inhibition of group I intron splicing in *Candida albicans* correlates with growth inhibition. Antimicrob. Agents Chemother. 44, 958–966. 10.1128/AAC.44.4.958-966.2000 10722497 PMC89798

[B52] Nawrocki E. P. Eddy S. R. (2013). Infernal 1.1: 100-fold faster RNA homology searches. Bioinformatics 29, 2933–2935. 10.1093/bioinformatics/btt509 24008419 PMC3810854

[B53] Nawrocki E. P. Burge S. W. Bateman A. Daub J. Eberhardt R. Y. Eddy S. R.. (2015). Rfam 12. 0 : updates to the RNA families database. Nucleic Acids Res. 43, 130–137. 10.1093/nar/gku1063 25392425 PMC4383904

[B54] Park I. K. Kim J. Y. (2001). NAD^+^ Inhibits the self-splicing of the group I intron. Biochem. Biophys. Res. Commun. 281, 206–211. 10.1006/bbrc.2001.4314 11178981

[B55] Rambaut A. (2009). FigTree version 1.4.2.

[B56] Robbins J. B. Smith D. Belfort M. (2011). Redox-responsive zinc finger fidelity switch in homing endonuclease and intron promiscuity in oxidative stress. Curr. Biol. 21, 243–248. 10.1016/j.cub.2011.01.008 21256016 PMC3621118

[B57] Rogozin I. B. Carmel L. Csuros M. Koonin E. V. (2012). Origin and evolution of spliceosomal introns. Biol. Direct 7, 1. 10.1186/1745-6150-7-11 22507701 PMC3488318

[B58] Ronquist F. Teslenko M. Van Der Mark P. Ayres D. L. Darling A. Höhna S.. (2012). Mrbayes 3.2: efficient bayesian phylogenetic inference and model choice across a large model space. Syst. Biol. 61, 539–542. 10.1093/sysbio/sys029 22357727 PMC3329765

[B59] Santos W. R. A. Meyer W. Wanke B. Evangelista Costa S. P. S. Trilles L. Nascimento J. L. M.. (2008). Primary endemic *Cryptococcosis gattii* by molecular type VGII in the state of Pará, Brazil. Mem. Inst. Oswaldo Cruz 103, 813–818. 10.1590/S0074-02762008000800012 19148422

[B60] Singh N. (2001). Trends in the epidemiology of opportunistic fungal infections: predisposing factors and the impact of antimicrobial use practices. Clin. Infect. Dis. 33, 1692–1696. 10.1086/323895 11641825

[B61] Sorrell T. C. (2001). Cryptococcus neoformans variety gattii. Med. Mycol. 39, 155–168. 10.1080/mmy.39.2.155.168 11346263

[B62] StataCorp (2017). Stata Statistical Software: Release 15. College Station, TX: StataCorp LLC.

[B63] Stoddard B. L. (2011). Homing endonucleases: from microbial genetic invaders to reagents for targeted DNA modification. Structure 19, 7–15. 10.1016/j.str.2010.12.003 21220111 PMC3038549

[B64] Trilles L. Lazéra S. Wanke B. Oliveira R. V. Barbosa G. G. Nishikawa M. M.. (2008). Regional pattern of the molecular types of *Cryptococcus neoformans* and *Cryptococcus gattii* in Brazil. Mem. Inst. Oswaldo Cruz. 103, 455–462. 10.1590/S0074-02762008000500008 18797758

[B65] Trilles L. Meyer W. Wanke B. Guarro J. Lazéra M. (2012). Correlation of antifungal susceptibility and molecular type within the *Cryptococcus neoformans*/*C. gattii* species complex. Med. Mycol. 50, 328–332. 10.3109/13693786.2011.602126 21859388

[B66] Yamamoto Y. Kohno S. Koga H. Kakeya H. Tomono K. Kaku M.. (1995). Random amplified polymorphic DNA analysis of clinically and environmentally isolated Cryptococcus neoformans in Nagasaki. J. Clin. Microbiol. 33, 3328–3332. 8586730 10.1128/jcm.33.12.3328-3332.1995 PMC228701

[B67] Zhang Y. Li Z. Pilch D. S. Leibowitz M. J. (2002). Pentamidine inhibits catalytic activity of group I intron Ca.LSU by altering RNA folding. Nucleic Acids Res. 30, 2961–2971. 10.1093/nar/gkf394 12087182 PMC117049

[B68] Zuker M. (2003). Mfold web server for nucleic acid folding and hybridization prediction. Nucleic Acids Res. 31, 3406–3415. 10.1093/nar/gkg595 12824337 PMC169194

