# Supplementary material for: Polymorphism in Mitochondrial Group I Introns among Cryptococcus neoformans and Cryptococcus gattii Genotypes and Its Association with Drug Susceptibility
Source: Front Microbiol. 2018 Feb 6;9:86. doi: 10.3389/fmicb.2018.00086 (PMC5808193; doi:10.3389/fmicb.2018.00086)
Supplement: Supplementary file 1 [file Table1.PDF]

Table S1: Genotyping and mitochondrial *LSU* rRNA introns occurrence in the isolates used in this work.

URA5-RFLP	Isolate ID	Isolate origin	*Amplicon* (bp)	Intron length (bp)			
				2439	2449	2504	2584
VNI	BT1	HC-SP	~300	-	-	-	-
VNI	BT2	HC-SP	~300	-	-	-	-
VNI	BT3	HC-SP	~300	-	-	-	-
VNI	BT4	HC-SP	~300	-	-	-	-
VNI	BT5	HC-SP	~300	-	-	-	-
VNI	BT6	HC-SP	~300	-	-	-	-
VNI	BT7	HC-SP	~300	-	-	-	-
VNI	BT9	HC-SP	~300	-	-	-	-
VNI	BT10	HC-SP	~300	-	-	-	-
VNI	BT11	HC-SP	~300	-	-	-	-
VNI	BT12	HC-SP	~1300	-	-	1032	-
VNI	BT13	HC-SP	~300	-	-	-	-
VNI	BT15	HC-SP	~300	-	-	-	-
VNI	BT16	HC-SP	~300	-	-	-	-
VNI	BT18	NI	~300	-	-	-	-
VNI	BT22	HV-SP	~300	-	-	-	-
VNI	BT23	HV-SP	~300	-	-	-	-
VNI	BT25	HV-SP	~300	-	-	-	-
VNI	BT26	HV-SP	~300	-	-	-	-
VNI	HGT1	HGT-RN	~300	-	-	-	-
VNI	HGT3	HGT-RN	~300	-	-	-	-
VNI	HGT6	HGT-RN	~300	-	-	-	-
VNI	HGT7	HGT-RN	~300	-	-	-	-
VNI	HGT8	HGT-RN	~300	-	-	-	-
VNI	HGT9	HGT-RN	~300	-	-	-	-
VNI	HGT11	HGT-RN	~300	-	-	-	-
VNI	HGT12	HGT-RN	~300	-	-	-	-
VNI	HGT13	HGT-RN	~300	-	-	-	-
VNI	HGT15	HGT-RN	~300	-	-	-	-
VNI	HGT16	HGT-RN	~1300	-	-	1032	-
VNI	UFRN1**	EI-RN	~1300	-	-	1032	-
VNI	UFRN2**	EI-RN	~300	-	-	-	-
VNI	PI1543	UFPI-PI	~300	-	-	-	-
VNI	PI1560	UFPI-PI	~300	-	-	-	-
VNI	CN772	IMT-SP	~300	-	-	-	-
VNI	CN216	IMT-SP	~1300	-	-	1032	-
VNI	LCR2002237	LACEN-RN	~300	-	-	-	-
VNI	LCR2002368	LACEN-RN	~1300	-	-	1032	-
VNI	CFP55	UFRGS-RS	~300	-	-	-	-
VNII	FC5 (WM626)*	WM	~1300	1073	-	-	-
VNII	HGT2	HGT-RN	~1300	1073	-	-	-
VNII	HGT4	HGT-RN	~1300	1073	-	-	-
VNII	CFP56	UFRGS-RS	~1300	1073	-	-	-
VNIII	FC4 (WM628)*	WM	~2500	925	579	417	-

VNIII	CN117	IMT-SP	~2000	-	1169	417	250
VNIII	CFP57	UFRGS-RS	~2000	>612	>292	417	250
VNIV	BT28	ATCC28958	~300	-	-	-	-
VNIV	BT29	ATCC28957	~300	-	-	-	-
VNIV	FC2 (WM629)*	WM	~1900	-	1169	417	250
VNIV	FC7	FIOCRUZ-RJ	~1900	716	578	-	-
VNIV	CFP58	UFRGS-RS	~1900	-	1168	417	250
VGI	BT14	HC-SP	~300	-	-	-	-
VGI	CFP59	UFRGS-RS	~1100	346	-	344	241
VGII	BT8	HC-SP	~300	-	-	-	-
VGII	BT17	HC-SP	~300	-	-	-	-
VGII	BT19	HV-SP	~300	-	-	-	-
VGII	BT20	HV-SP	~300	-	-	-	-
VGII	BT21	HV-SP	~300	-	-	-	-
VGII	BT24	HV-SP	~300	-	-	-	-
VGII	BT27	NI	~300	-	-	-	-
VGII	FC1 (WM178)*	WM	~300	-	-	-	-
VGII	FC6	FIOCRUZ-RJ	~300	-	-	-	-
VGII	HGT5	HGT-RN	~300	-	-	-	-
VGII	HGT10	HGT-RN	~300	-	-	-	-
VGII	HGT14	HGT-RN	~300	-	-	-	-
VGII	HSL1	HSL-RN	~300	-	-	-	-
VGII	PI1401	UFPI-PI	~300	-	-	-	-
VGII	CG606	IMT-SP	~300	-	-	-	-
VGII	CG201	IMT-SP	~300	-	-	-	-
VGII	CG751	IMT-SP	~300	-	-	-	-
VGII	CG769	IMT-SP	~300	-	-	-	-
VGII	CN894	IMT-SP	~300	-	-	-	-
VGII	CN508	IMT-SP	~300	-	-	-	-
VGIII	FC3 (WM161)*	WM	~1800	1059	-	-	-
VGIII	CFP61	UFRGS-RS	~1800	1073	-	390	252
VGIV	FC9 (WM779)*	FIOCRUZ-RJ	~1300	1059	-	-	-
VGIV	CFP62	UFRGS-RS	~1300	1059	-	-	-
NA	FC8 (*C. laurentii)*	FIOCRUZ-RJ	NA	NA	NA	NA	NA

Legend: CFP57 isolate (VNIII genotype) was incompletely sequenced. Cne.mL2439 and Cne.mL2449 had 612 and 292 nucleotides sequenced, respectively. NI – isolates with uninformed origin (from mycological collection of IBB-UNESP, Botucatu, São Paulo State, Brazil); NA - not applicable; HC - Botucatu Clinic hospital/UNESP, Botucatu, São Paulo State, Brazil; HV - Botucatu Veterinary hospital/UNESP, Botucatu, São Paulo State, Brazil; HGT - Giselda Trigueiro Hospital, Natal, Rio Grande do Norte State, Brazil; UFPI - Federal University of Piauí, Piauí State, Brazil; IMT-SP - Institute of Tropical Medicine of São Paulo, São Paulo, São Paulo State, Brazil; LACEN-RN, Central Laboratory of Rio Grande do Norte, Natal, Rio Grande do Norte State, Brazil; UFRGS-RS - Federal University of Rio Grande do Sul, Porto Alegre, Rio Grande do Sul State, Brazil; FIOCRUZ-RJ – Fundação Oswaldo Cruz, Rio de Janeiro, Rio de Janeiro State, Brazil; HSL-RN - São Lucas Hospital, Natal, Rio Grande do Norte State, Brazil; -: intron absence; NS - not sequenced; *WM – from Wieland Meyer collection, University of Sydney, Australia (Meyer, et al. 2003); ** EI- Environmental Isolate from pigeons feces, Alecrim, Natal, Rio Grande do Norte State, Brazil.
