# Supplementary material for: Polymorphism in Mitochondrial Group I Introns among Cryptococcus neoformans and Cryptococcus gattii Genotypes and Its Association with Drug Susceptibility
Source: Front Microbiol. 2018 Feb 6;9:86. doi: 10.3389/fmicb.2018.00086 (PMC5808193; doi:10.3389/fmicb.2018.00086)
Supplement: Supplementary file 2 [file Table2.PDF]

Table S2. E-value and score obtained by Infernal v1.1.2 [38] for the alignment of the introns Cne.mL2439/Cga.mL2439 and Cne.mL2584/Cga.mL2584 with the RF00028 [39] group I intron model.

**Intron**	**E-value**	**Score**
**Cga.mL2439_CFP59_VGI**	9,8E-17	62,6
**Cga.mL2439_CFP61_VGIII**	1,7E-17	65,3
**Cga.mL2439_CFP62_VGIV**	2,1E-17	64,9
**Cga.mL2439_FC3_VGIII**	2,1E-17	64,9
**Cga.mL2439_FC9_VGIV**	2,1E-17	64,9
**Cne.mL2439_CFP56_VNII**	6,5E-17	63,2
**Cne.mL2439_DQ479323.1_VNIII**	1,3E-15	58,7
**Cne.mL2439_FC4_VNIII**	2,5E-16	61,2
**Cne.mL2439_FC5_VNII**	6,5E-17	63,2
**Cne.mL2439_FC7_VNIV**	2,5E-16	61,2
**Cne.mL2439_HGT2_VNII**	6,5E-17	63,2
**Cne.mL2439_HGT4_VNII**	6,5E-17	63,2
**Cne.mL2449_AY560611.1_VNIV**	9,1E-18	66,2
**Cne.mL2449_CFP58_VNIV**	9,1E-18	66,2
**Cne.mL2449_CN117_VNIII**	1,5E-19	72,4
**Cne.mL2449_DQ479323.1_VNIII**	4,8E-17	63,7
**Cne.mL2449_FC2_VNIV**	9,9E-20	73
**Cne.mL2449_FC4_VNIII**	4,8E-17	63,7
**Cne.mL2449_FC7_VNIV**	5,1E-19	70,5
**Cne.mL2504_AY560611.1_VNIV**	3,1E-17	64,3
**Cne.mL2504_BT12_VNI**	1,2E-15	58,8
**Cne.mL2504_CFP57_VNIII**	1,3E-16	62,2
**Cne.mL2504_CFP58_VNIV**	3,1E-17	64,3
**Cne.mL2504_CFP59_VGI**	1,7E-19	72,2
**Cne.mL2504_CFP61_VGIII**	4,6E-14	53,3
**Cne.mL2504_CN117_VNIII**	4,3E-12	46,5
**Cne.mL2504_CN216_VNI**	1,2E-15	58,8
**Cne.mL2504_DQ479323.1_VNIII**	1,3E-16	62,2
**Cne.mL2504_FC2_VNIV**	3,1E-17	64,3
**Cne.mL2504_FC4_VNIII**	1,3E-16	62,2
**Cne.mL2504_HGT16_VNI**	1,2E-15	58,8
**Cne.mL2504_LCR2002368_VNI**	1,2E-15	58,8
**Cne.mL2504_UFRN1_VNI**	1,2E-15	58,8
**Cne.mL2584 _CFP57_VNIII**	4,5E-16	60,3
**Cne.mL2584_CFP58_VNIV**	1,9E-15	58,2
**Cga.mL2584_CFP59_VGI**	7,6E-16	59,5
**Cga.mL2584_CFP61_VGIII**	1,2E-14	55,3
**Cne.mL2584_CN117_VNIII**	1,9E-15	58,2
**Cne.mL2584_FC2_VNIV**	1,9E-15	58,2
