# Supplementary material for: Polymorphism in Mitochondrial Group I Introns among Cryptococcus neoformans and Cryptococcus gattii Genotypes and Its Association with Drug Susceptibility
Source: Front Microbiol. 2018 Feb 6;9:86. doi: 10.3389/fmicb.2018.00086 (PMC5808193; doi:10.3389/fmicb.2018.00086)
Supplement: Supplementary file 3 [file Table3.PDF]

Table S3. Genbank accession numbers of intron I sequences used in phylogenetic analysis of the LSU introns from *Cryptococcus*.

Genbank accession number	Species	Host gene
AB105924.1	*Cryptococcus gattii*	*COB*
AB105931.1	*Cryptococcus gattii*	*COB*
AB175758.1	*Trichosporon faecale*	*COB*
AB175776.1	*Trichosporon ovoides*	*COB*
AF394207.1	*Polytoma_oviforme*	*16s (*Leucoplast)
AF538048.1	*Amoebidium parasiticum*	*NAD5*
AY781064.1	*Agrocybe aegerita*	*COB*
AY863212.1	*Rhizopus oryzae*	*NAD3*
AY863212.1	*Rhizopus oryzae*	*COB*
AY863212.1	*Rhizopus oryzae*	*ATP9*
DQ157700.1	*Ustilago maydis*	*COX1*
DQ157700.1	*Ustilago maydis*	*NAD5*
DQ209280.1	*Uromyces appendiculatus*	*COB*
EU921808.1	*Ustilago maydis*	*LSU*
FN356025.1	*Pichia farinosa*	*LSU*
FQ311469.1	*Sporisorium reilianum*	*COB*
FQ311469.1	*Sporisorium reilianum*	*NAD5*
FQ311469.1	*Sporisorium reilianum*	*COX1*
GQ354525.2	*Brettanomyces custersianus*	*COX1*
HE983614.1	*Lachancea meyersii*	*LSU*
HF546977.1	*Rhizoctonia solani*	Mitochondrial DNA sequence
JQ062883.1	*Candida parapsilosis*	*LSU*
JX271275.1	*Agaricus bisporus*	*LSU*
JX985789.1	*Tricholoma matsutake*	Mitochondrial DNA sequence
KC164355.1	*Glomus_sp*	*COB*
KC285587.1	*Microbotryum violaceum*	*LSU*
KC763799.1	*Ganoderma lucidum*	*COX1*
KC993181.1	*Cyberlindnera suaveolens*	*LSU*
KF017574.1	*Candida chauliodes*	*LSU*
KF423149.1	*Bullera koratensis*	*COB*
KF423212.1	*Holtermanniella nyarrowii*	*COB*
KF431978.1	*Puccinia psidii*	*COX1*
KF673550.1	*Ganoderma sinense*	*COX1*
KJ806271.1	*Neochloris aquatica*	*LSU*
KM382275.1	*Parasitella parasitica*	*COB*
KM382275.1	*Parasitella parasitica*	*ATP9*
KM382275.1	*Parasitella parasitica*	*LSU*
KP323060.1	*Cintractia sorghi-vulgaris*	*COB*
KR109212.1	*Ganoderma applanatum*	*NAD5*
KR809878.1	*Phycomyces blakesleeanus*	*COB*
KR809878.1	*Phycomyces blakesleeanus*	*LSU*
KU162859.1	*Rhizophagus irregularis*	*COB*
KU196782.1	*Absidia glauca*	*ATP9*
LT558140.1	*Ustilago bromivora*	*ATP9*
KC993176.1	*Meyerozyma guilliermondii*	*NAD5*

All sequences obtained in blastn tool share some similarity with group I introns here sequenced. Sequences with query cover lower than 25% were not selected.
