# Supplementary material for: Polymorphism in Mitochondrial Group I Introns among Cryptococcus neoformans and Cryptococcus gattii Genotypes and Its Association with Drug Susceptibility
Source: Front Microbiol. 2018 Feb 6;9:86. doi: 10.3389/fmicb.2018.00086 (PMC5808193; doi:10.3389/fmicb.2018.00086)
Supplement: Supplementary file 4 [file Table4.PDF]

**S4 Table.** MIC observed for *C. neoformans* and *C. gattii* isolates from different genotypes presenting polymorphisms concerning the introns in mitochondrial *LSU* rRNA.

| *Amplicon* | Intron lenght (bp) | | | | Genotypes | Isolate | MICs values (µg/mL) | | |
|---|---|---|---|---|---|---|---|---|---|
| | 2439 | 2449 | 2504 | 2584 | | | Amphotericin | Itraconazole | 5-fluorocytosine |
| 300bp | - | - | - | - | VNI | UFRN2 | 0,5 | 0,03125 | 16 |
| | - | - | - | - | VNI | CN772 | 0,25 | 0,125 | 4 |
| | - | - | - | - | VNI | PI1543 | 0,25 | 0,03125 | 16 |
| | - | - | - | - | VNI | HGT03 | 0,125 | 0,03125 | 8 |
| | - | - | - | - | VNI | HGT06 | 0,25 | 0,03125 | 2 |
| | - | - | - | - | VNI | HGT07 | 0,25 | 0,03125 | 16 |
| | - | - | - | - | VNI | HGT08 | 0,25 | 0,03125 | 8 |
| | - | - | - | - | VNI | HGT13 | 0,25 | 0,03125 | 16 |
| | - | - | - | - | VNIV | BT28 | 0,125 | 0,03125 | 4 |
| | - | - | - | - | VNIV | BT29 | 0,125 | 0,03125 | 2 |
| | - | - | - | - | VGI | BT14 | 0,25 | 0,0625 | 8 |
| | - | - | - | - | VGII | HGT05 | 0,125 | 0,03125 | 8 |
| | - | - | - | - | VGII | BT27 | 0,0625 | 0,03125 | 16 |
| | - | - | - | - | VGII | HGT10 | 0,125 | 0,25 | 2 |
| | - | - | - | - | VGII | FC6 | 0,125 | 0,03125 | 4 |
| | - | - | - | - | VGII | PI1401 | 0,0625 | 0,03125 | 8 |
| | - | - | - | - | VGII | CG769 | 0,125 | 0,03125 | 2 |
| | - | - | - | - | VGII | BT21 | 0,125 | 0,0625 | 16 |
| | - | - | - | - | VGII | HGT14 | 0,25 | 0,25 | 8 |
| | - | - | - | - | VGII | HSL1 | 0,125 | 0,03125 | 16 |
| 1,1Kb | 346 | - | 344 | 241 | VGI | CFP59 | 0,25 | 0,03125 | 2 |
| 1,3Kb | - | - | 1032 | - | VNI | BT12 | 0,25 | 0,03125 | 8 |
| | - | - | 1032 | - | VNI | UFRN1 | 0,25 | 0,03125 | 4 |
| | - | - | 1032 | - | VNI | CN216 | 0,125 | 0,0625 | 4 |
| | - | - | 1032 | - | VNI | HGT16 | 0,25 | 0,03125 | 4 |
| | - | - | 1032 | - | VNI | LCR2002368 | 0,5 | 0,03125 | 4 |
| | 1073 | - | - | - | VNII | FC5 | 0,125 | 0,125 | 8 |
| | 1073 | - | - | - | VNII | HGT02 | 0,25 | 0,03125 | 8 |
| | 1073 | - | - | - | VNII | HGT04 | 0,5 | 0,03125 | 2 |
| | 1073 | - | - | - | VNII | CFP56 | 0,25 | 0,03125 | 8 |
| | 1059 | - | - | - | VGIV | FC9 | 0,25 | 0,25 | 4 |
| | 1059 | - | - | - | VGIV | CFP62 | 0,25 | 0,25 | 4 |
| 1,8Kb | 1059 | - | - | - | VGIII | FC3 | 0,25 | 0,03125 | 4 |
| | 1073 | - | 390 | 252 | VGIII | CFP61 | 0,25 | 0,03125 | 2 |
| 1,9Kb | - | 1169 | 417 | 250 | VNIV | FC2 | 0,125 | 0,03125 | 16 |
| | 716 | 578 | - | - | VNIV | FC7 | 0,125 | 0,03125 | 8 |
| | - | 1168 | 417 | 250 | VNIV | CFP58 | 0,125 | 0,03125 | 8 |
| 2,0Kb | - | 1169 | 417 | 250 | VNIII | CN117 | 0,0625 | 0,03125 | 8 |
| | >612 | >292 | 417 | 250 | VNIII | CFP57 | 0,125 | 0,03125 | 4 |
| 2,5Kb | 925 | 579 | 417 | - | VNIII | FC4 | 0,125 | 0,03125 | 8 |
