# Supplementary figures and images for: Polymorphism in Mitochondrial Group I Introns among Cryptococcus neoformans and Cryptococcus gattii Genotypes and Its Association with Drug Susceptibility
Source: Front Microbiol. 2018 Feb 6;9:86. doi: 10.3389/fmicb.2018.00086 (PMC5808193; doi:10.3389/fmicb.2018.00086)

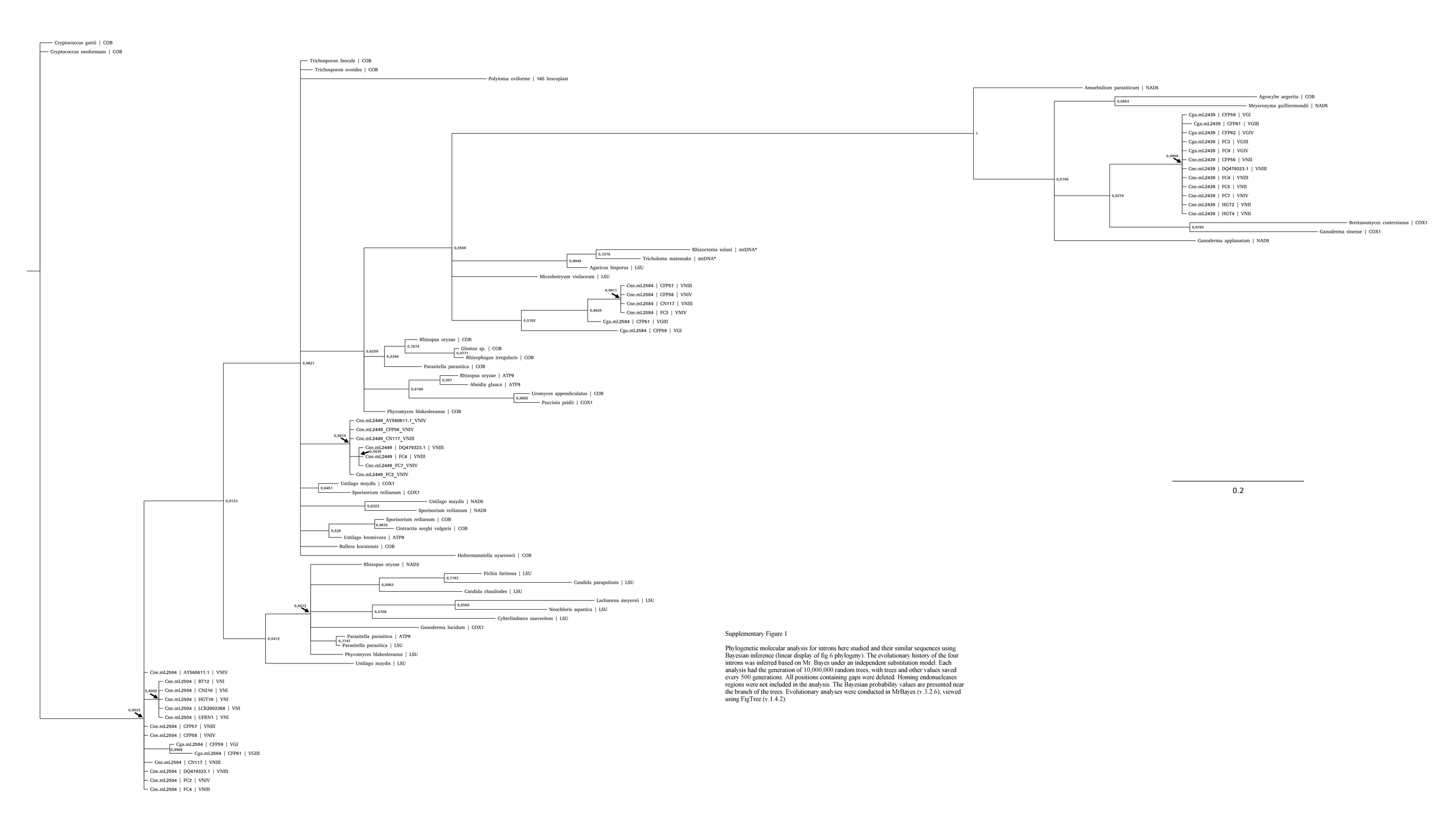

Supplement: Supplementary file 6 [file Image1.TIFF]
